# Supplementary material for: Cryo-EM structure of the human Kv3.1 channel reveals gating control by the cytoplasmic T1 domain
Source: Nat Commun. 2022 Jul 15;13:4087. doi: 10.1038/s41467-022-29594-w (PMC9287412; doi:10.1038/s41467-022-29594-w)
Supplement: Supplementary file 1 — Supplementary Information [file 41467_2022_29594_MOESM1_ESM.pdf]

Supplementary Information for

Cryo-EM structure of the human Kv3.1 channel reveals gating control by the cytoplasmic T1 domain

Gamma Chi[1,2], Qiansheng Liang[3], Akshay Sridhar[4], John B. Cowgill[4], Kasim Sader[5], Mazdak Radjainia[5], Pu Qian[5], Pablo Castro-Hartmann[5], Shayla Venkaya[1,2,6], Nanki Kaur Singh[1,2], Gavin McKinley[1,2], Alejandra Fernandez-Cid[1,2,7], Shubhashish M. M. Mukhopadhyay[1,2,6], Nicola A. Burgess-Brown[1,2,7], Lucie Delemotte[4], Manuel Covarrubias[3] and Katharina L. Dürr[1,2,8,‡]

[1] Centre for Medicines Discovery, Nuffield Department of Medicine, University of Oxford, *Roosevelt* Drive, Oxford, OX3 7DQ, UK

[2] Structural Genomics Consortium, Nuffield Department of Medicine, University of Oxford, *Roosevelt* Drive, Oxford, OX3 7DQ, UK

[3] Department of Neuroscience and Vickie and Jack Farber Institute for Neuroscience, Sidney Kimmel Medical College at Thomas Jefferson University, Philadelphia, PA 19107

[4] Department of Applied Physics, Science for Life Laboratory, KTH, Solna, Sweden

[5] Materials and Structural Analysis, Thermo Fisher Scientific, Achtseweg Noord 5, 5651 GG Eindhoven, Netherlands

[6] Present address: Exscientia Ltd., The Schrödinger Building, Heatley Road, The Oxford Science Park, Oxford, OX4 4GE

[7] Present address: Exact Sciences Ltd., The Sherard Building, Edmund Halley Road, The Oxford Science Park, Oxford, OX4 4DQ

[8] Present address: OMass Therapeutics, Ltd., The Schrödinger Building, Heatley Road, The Oxford Science Park, Oxford, OX4 4GE, UK

[‡] correspondence should be addressed to: katharina.duerr@cmd.ox.ac.uk

Table of Contents

Suppl. Figure S1. *Sequence alignment of human Kv3.1a with other human Kv channels………………………...3*

Suppl. Table 1. *Data acquisition and processing parameters and model refinement statistics for Kv3.1a datasets collected under apo, Zn^{2+}-free and Zn^{2+}-containing conditions…………………………………………………..5*

Suppl. Figure S2. *Purification and cryo-EM analysis of Kv3.1a under apo conditions……………..………………..6*

Suppl. Figure S3. *Cryo-EM data processing of Kv3.1a in presence of EDTA or $ZnCl_2$…………………..……………….7*

Suppl. Figure S4. *EM density maps for representative segments of Kv3.1a……………………………………………..8*

Suppl. Figure S5. *Detailed mapping of disease mutations in the human KCNC1 gene linked to EPM7 and related epileptic encephalopathies………………………………………………………………………………………………………9*

Suppl. Figure S6. *T1 domain superposition of Kv3.1a with Kv1.3 (+β2.1) and Kv1.2-2.1………………………….10*

Suppl. Figure S7. *C-terminal extension (ATM) interacts with two adjacent subunits from T1…………………11*

Suppl. Figure S8. *Comparison of the Kv3.1a cryo-EM structure to model predicted by Alpha-Fold2 shows differences in the arrangement of S4 and the S4/S5 linker……………………………………………………………..…12*

Suppl. Figure S9. *Comparison of electrostatic surface potential distribution for human Kv channel structures of Kv3.1a, Kv1.2-2.1 and Kv1.3…………………………………………….……………………………………..……13*

Suppl. Figure S10. *Comparison of electrostatic surface charges near α4 in T1 of Kv3.1a and in T1 domains from representative channels of the Kv1 and Kv4 subfamilies…………………………………………………….……14*

Suppl. Figure S11. *Pore hydration properties and characterization of the D81K mutant by MD simulations and TEVC recordings in Xenopus oocytes…………………………………………………………………………………………….15*

Suppl. Figure S12. *Comparison of extra- and intracellular linker arrangement in the voltage-sensing domains of Kv3.1a and Kv1.2-2.1 and VSD arrangement in the Kv4.2 structure…………………………………..16*

Suppl. Figure S13. *TEVC recordings of Kv3.1a channels in Xenopus oocytes…………………………………………17*

Suppl. Figure S14. *Activation and deactivation time constants for mutant and wildtype Kv3.1…………….18*

Suppl. Figure S15. *Z-position trajectories of the S4 gating charges R1-R4 relative to the CTC in Kv3.1a for activating and deactivating voltages…………………………………………………………………………………………...19*

Suppl. Figure S16. *Analysis of lipid/protein interactions in the human Kv3.1a channel………………………..20*

Suppl. Figure S17. *Illustration of dimer interface interactions for a subset of particles forming Kv3.1a dimers in the dataset with 400 μM $ZnCl_2$…………………………………………………………………………………21*

Suppl. Table 2. *Summary of best-fit Boltzmann function parameters of G-V curves obtained for wildtype and mutant Kv3.1a channels……………………………………………………………………………………………………..22*

Supplementary results and discussion…………………………………………………………………………………………23

Supplementary methods……..25

References…….26

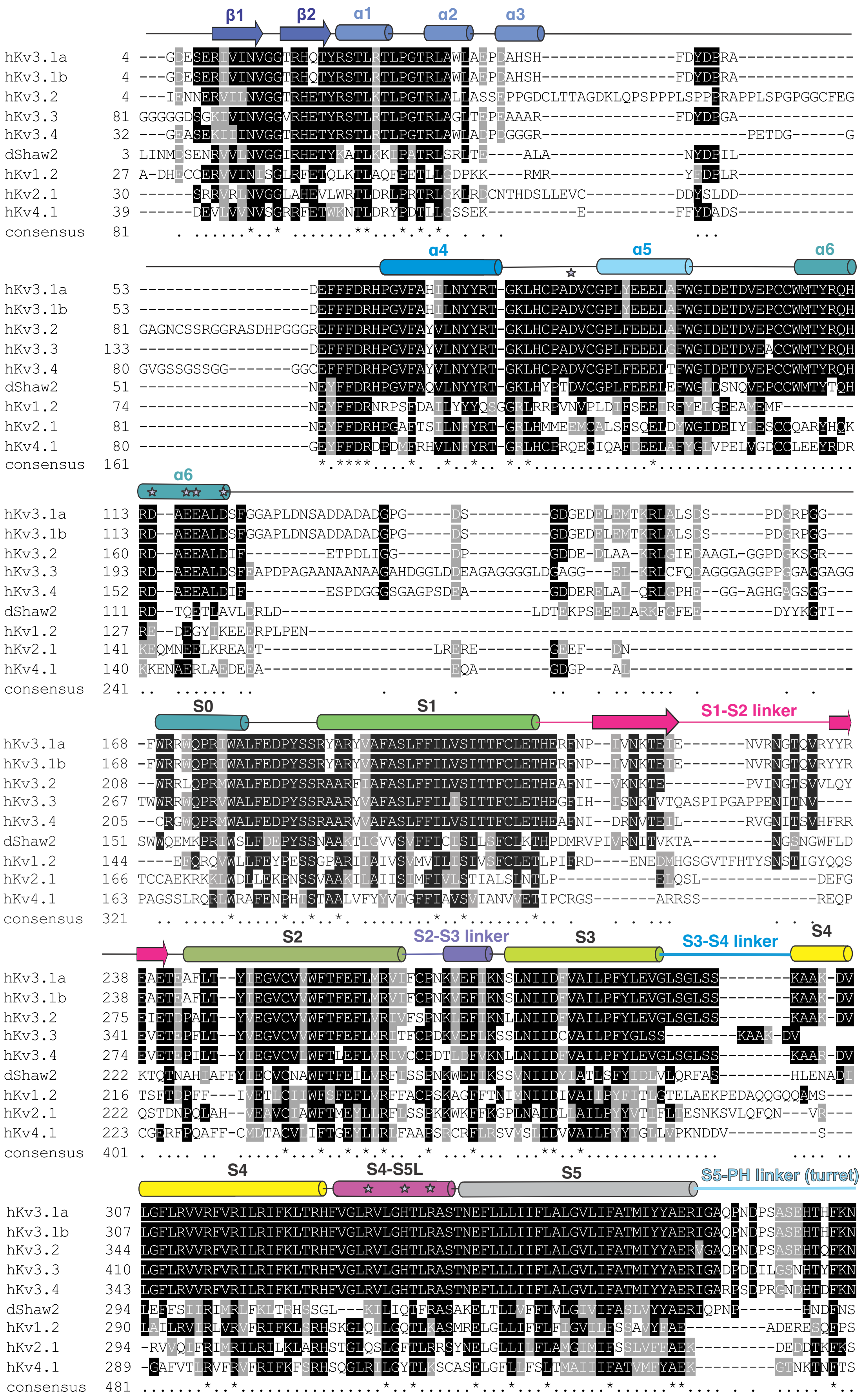
β1
β2
α1
α2
α3
α4
α5
α6
S0
S1
S1-S2 linker
S2
S2-S3 linker
S3
S3-S4 linker
S4
S4-S5L
S5
S5-PH linker (turret)
hKv3.1a
hKv3.1b
hKv3.2
hKv3.3
hKv3.4
dShaw2
hKv1.2
hKv2.1
hKv4.1
consensus

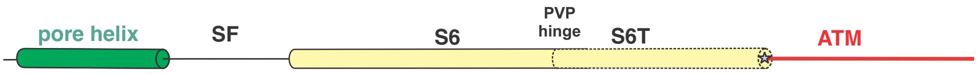


```
hKv3.1a     387  IPIGFWWAVVTMTTLGYGDMYPQTWSGMLVGALCALAGVLTIAMPVPVIVNNFGMYYSLAMAKQKLPKKKKKHIP-----
hKv3.1b     387  IPIGFWWAVVTMTTLGYGDMYPQTWSGMLVGALCALAGVLTIAMPVPVIVNNFGMYYSLAMAKQKLPKKKKKHIP-----
hKv3.2      424  IPIGFWWAVVTMTTLGYGDMYPQTWSGMLVGALCALAGVLTIAMPVPVIVNNFGMYYSLAMAKQKLPRKRKKHIP-----
hKv3.3      490  IPIGFWWAVVTMTTLGYGDMYPKTWSGMLVGALCALAGVLTIAMPVPVIVNNFGMYYSLAMAKQKLPKKKNKHIP-----
hKv3.4      423  IPIGFWWAVVTMTTLGYGDMYPKTWSGMLVGALCALAGVLTIAMPVPVIVNNFGMYYSLAMAKQKLPKKRKKHVP-----
dShaw2      364  IPLGLWWALVTMTTVGYGDMAPKTYIGMFVGALCALAGVLTIALPVPVIVSNFAMYYSHTQARAKLPKKRRRVLPVEQPR
hKv1.2      361  IPDAFWWAVVSMTTVGYGDMVPTTIGGKIVGSLCAIAGVLTIALPVPVIVSNFNYFYH----RETEGEEQAQYLQVTSC-
hKv2.1      364  IPASFWWATITMTTVGYGDIYPKTLLGKIVGGLCCIAGVLVIALPIPIIVNNFSEFYKEQKRQEKAIKRRE---------
hKv4.1      359  IPAAFWYTIVTMTTLGYGDMVPSTIAGKIFGSICSLSGVLVIALPVPVIVSNFSRIYHQNQRADKRRAQQKVRLARIRLA
consensus    561    **...*.....***.****..*   *..*...*..*...***.**.*.*.**.**...*..................
```

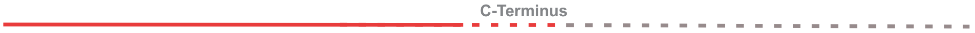


```
hKv3.1a     462  RP-----------------------------------PQLGSPNYCKSVVNS-----PH-----------HS---T----
hKv3.1b     462  RP-----------------------------------PQLGSPNYCKSVVNS-----PH-----------HS---T----
hKv3.2      499  PA-----------------------------------PQASSPTFCKTELNM-----AC-----------NS---T----
hKv3.3      565  RP-----------------------------------PQPGSPNYCKPDPPPPPPPHPH-----------HG---SGGIS
hKv3.4      498  RP-----------------------------------AQLESPMYCKSEETS-----PR-----------DS---T----
dShaw2      444  QP-----------------------------------RLPGAPGGVSGCGTPGSG--PH-----------SGPMGSGGTG
hKv1.2      436  -P-----------------------------------KIPSSPDLKKSRSASTISKSDY-----------MEI--QEGVN
hKv2.1      435  ------ALERAKRNGSIVSMNMKDAFARSIEMMDIVVEKNGENMGKKDKVQDNHLSPNKWKWTKRTLSETSSSKSFETKE
hKv4.1      439  KSGTTNAFLQYKQNGGLEDSGSGEEQALCVRNRSAFEQQHHHLLHCLEKTTCHEFTD------ELTFSEALGAVSPGGRT
consensus   641 ..                                       . ...  ....          ..              .   .

hKv3.1a     484  ----------------------------------------------------------------QSDTCPLAQEE-ILEI
hKv3.1b     484  ----------------------------------------------------------------QSDTCPLAQEE-ILEI
hKv3.2      521  ----------------------------------------------------------------QSDTCL-GKDNRLLEH
hKv3.3      596  P------PPPITPPSMGVT-VAGAYPAGPHTHPGLLRGGAGGLGIMGLPPL-----------PAPGEPCPLAQEE-VIEI
hKv3.4      520  ----------------------------------------------------------------CSDTSPPAREEGMIER
dShaw2      476  PRRMNNKTKDLVSPKSDMA-FSFD--------------------------------------------------------
hKv1.2      467  NSNEDF--REENLKTANCT-LANTNYVNITKMLTDV--------------------------------------------
hKv2.1      509  QGSPEKARSSSSPQHLNVQQLEDMYNKMAKTQSQPILNTKESAAQSKPKE-----ELEMESIPSPVAPLPTRTEG-VIDM
hKv4.1      513  SRSTSVSSQPVGPGSL----LSSCCPRRAKRRAIRLANSTASVSRGSMQELDMLAGLRRSHAPQSRSSLNAKPHD-SLDL
consensus   721                                                                    .. . . .. ....

hKv3.1a     499  NRAGRK---------------------------PLRGMSI----------------------------------------
hKv3.1b     499  NRADSKLNGEVAKAALANEDCPHIDQAL----TPDEGLPFTRSGTRERY---GPCFLLSTGEYAC---------------
hKv3.2      536  NRS-----------VLSGDDSTGSEPPL----SPPERLPIRRSSTRDKNRRGETCFLLTTGDYTC---------------
hKv3.3      657  NRADPRPNGDPAAAALAHEDCPAIDQPAM---SPEDKSPIT-PGSRGRYSRDRACFLLT--DYAP---------------
hKv3.4      536  KRADSKQNGD-ANAVLSDEEGAGLTQPLASSPTPEERRALRRSTTRDRNKKAAACFLLSTGDYAC---------------
dShaw2           --------------------------------------------------------------------------------
hKv1.2           --------------------------------------------------------------------------------
hKv2.1      583  R-SMSSIDSFISCAT----DFPEATRFSH---SPLTSLPSK-TGGSTAPEVGWRGALGASGGRFVEANPSPDASQHSSFF
hKv4.1      588  N-CDSR--DFVAAII----SIP--TPPAN---TPDESQPSS-PGGGGRAGSTLRNSSLGTPCLFPETVKISSL-------
consensus   801 ..     .                   .                 .. .   .           .            .. .

hKv3.1a          --------------------------------------------------------------------------------
hKv3.1b     557  ------------------------------------------------------------------PPGGGM-RKDLCKE
hKv3.2      586  ------------------------------------------------------------------ASDGGI-RKGYEKS
hKv3.3      716  ------------------------------------------------------------------SPDGSI-RKAT-GA
hKv3.4      600  ------------------------------------------------------------------A-DGSV-RKGTFVL
dShaw2           --------------------------------------------------------------------------------
hKv1.2           --------------------------------------------------------------------------------
hKv2.1      654  IESPKSSMKTNNPLKLRALKVNFMEGDPSPLLPVLGMYHDPLRNRGSAAAAVAGLECATLLDKAVLSPESSIYTTASAKT
hKv4.1           --------------------------------------------------------------------------------
consensus   881                                                                                 .

hKv3.1a          --------------------------------------------------------------------------------
hKv3.1b     570  SP------------------------------------------V--IA-------------------------KYMPT-
hKv3.2      599  RS------------------------------------------LNNIAGLA-------------GNALRLSPVTSPYN-
hKv3.3      728  PP------------------------------------------LP-----P-------------QDWRKPGPPSFLPD-
hKv3.4      612  RD------------------------------------------L---------------------------PLQHSPE-
dShaw2           --------------------------------------------------------------------------------
hKv1.2           --------------------------------------------------------------------------------
hKv2.1      734  PPRSPEKHTAIAFNFEAGVHQYIDADTDDEGQLLYSVDSSPPKSLPGSTS-PKFSTGTRSEKNHFESSPLPTSPKFLRQN
hKv4.1           --------------------------------------------------------------------------------
consensus   961                                                                                 .
```

Supplemental Figure S1. Sequence alignment of human Kv3 family members Kv3.1-Kv3.4, Shaw2 (a Kv3 orthologue from *d. melanogaster*) and other representative channels from human Kv1-4 subfamilies.

The sequences were aligned with MUSCLE and presented with BOXSHADE. Grey shaded regions indicate areas of high sequence conservation, invariant amino acids are shaded in black. Secondary structure elements are color-coded as in the main figures of the manuscript. Star symbols indicate residues mutated for electrophysiological characterization in this study. The uniprot IDs for the aligned sequences are: hKv3.1a: P48547, hKv3.1b: P48547-2, hKv3.1: Q96PR1, hKv3.3: Q14003, hKv3.4:Q03721, dShaw2: P17972, hKv1.2: P16389, hKv2.1: Q14721, hKv4.1: Q9NSA2.

	KCNC1 (Apo)	KCNC1 with EDTA			KCNC1 with $ZnCl_2$	
**Data collection**						
Microscope	Titan Krios (MRCEF, UK)	Titan Krios (CNC, UK)			Titan Krios, (CNC, UK)	
Detector	K3	K3			K3	
Voltage (kV)	300	300			300	
Magnification	105,000	105,000			105,000	
Collection mode	Counting (superresolution)	Counting (superresolution)			Counting (superresolution)	
Electron exposure (e/Å^2)	40	47.6			47.6	
Number of frames	45	40			40	
Pixel size	0.42	0.42			0.42	
Defocus range (µm; steps)	-1.0 to -2.6 (0.2)	-0.6 to -2.4 (0.2)			-0.6 to -2.4 (0.2)	
Number of movies	4,043	7,274			5,010	
Phase plate used	No	No			No	
**Data processing**		**Consensus**	**Subclass 1**	**Subclass 2**	**Monomer**	**Dimer**
Initial Number of particles	1,539,146	3,177,434			2,672,854	
Number of particles after 2D classification	58,244	217,788			263,026	
Symmetry	C4	C4	C1	C1	C4	C4
Number of particles used for 3D refinement	49,327	217,788	110,585	93,461	133,488	72,764
Map resolution (Å; FSC threshold = 0.143)	3.2	3.2	3.6	3.6	3.1	3.1
Resolution range (Å)	2.7 – 9.3	2.6 – 6.4	3.0 – 8.7	3.5 – 8.4	2.7 – 8.0	2.6 – 9.5
Map sharpening B-factor (Å^2)	-134.4	-164.3	-135.0	-102	-153.2	-108.4
**Refinement**						
Model resolution (Å; FSC threshold = 0.5)	3.5	3.4			3.4	3.3
**Model composition**						
Non-hydrogen atoms	12,720	12,604			12,708	25,404
Protein residues	1584	1564			1,572	3140
Ligands	16	16			16	32
**R.M.S.D**						
Bond lengths (Å)	0.007	0.007			0.003	0.004
Bond angles (°)	0.700	0.672			0.494	0.530
**Validation**						
Molprobity score	1.61	1.57			1.64	1.70
Clash score	7.68	6.46			7.79	5.89
Rotamer outliers	0.96	0.96			0.00	2.53
**Ramachandran plot**						
Disallowed (%)	0.00	0.00			0.00	0.00
Allowed (%)	3.09	3.33			3.38	2.34
Favoured (%)	96.91	96.67			96.62	97.66
**EMDB Code**	EMD-13416	EMD-13419			EMD-13417	EMD-13418
**PDB Code**	7PHH	7PHL			7PHI	7PHK

Supplemental Table 1. Cryo-EM data collection and processing parameters and model refinement statistics for Kv3.1a datasets collected under apo, Zn^{2+}-free and Zn^{2+}-containing conditions at the Midlands Regional cryo-EM Facility (MRCEF) or at the Cambridge Nanocentre (CNC), respectively.

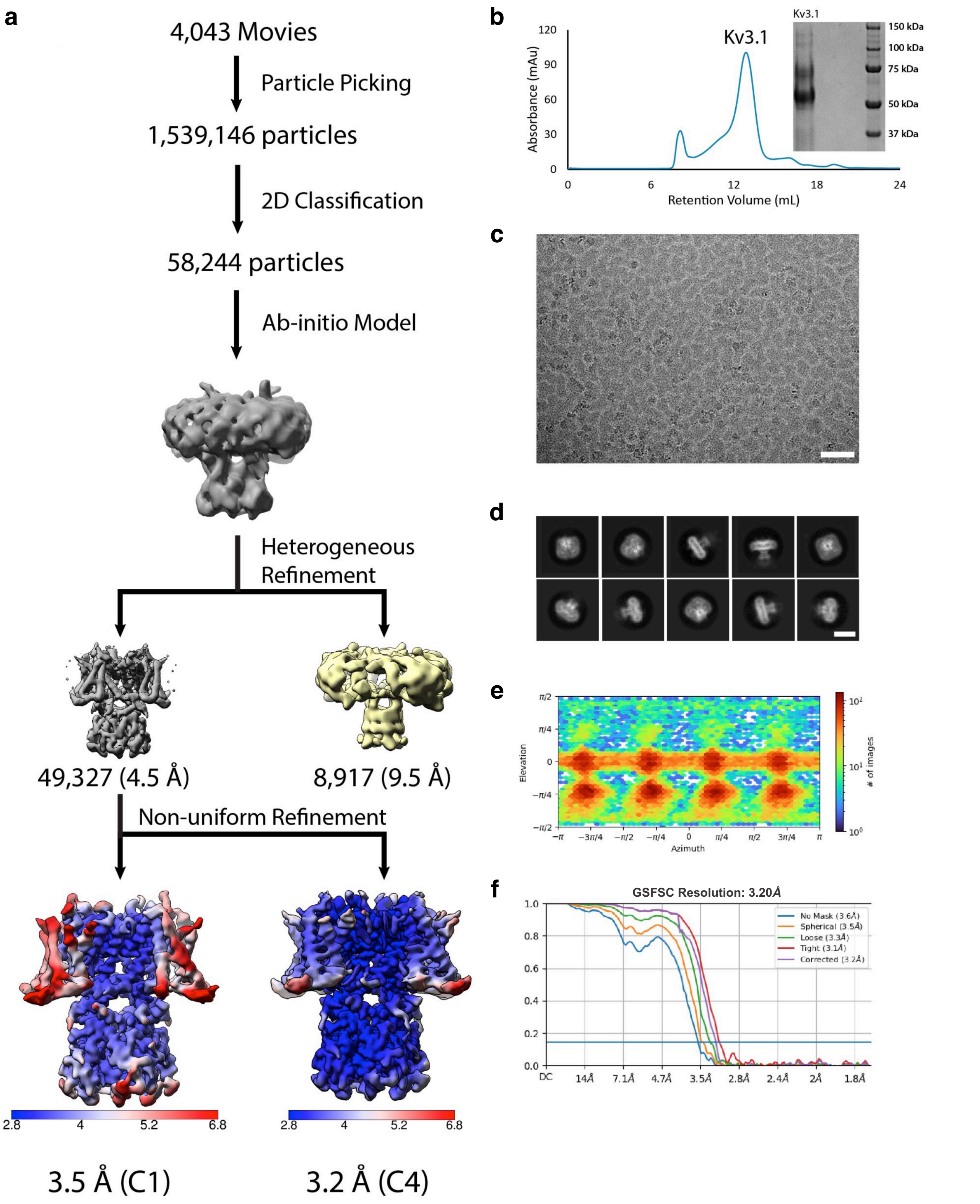

Supplemental Figure S2. Purification and cryo-EM analysis of Kv3.1a under apo conditions.

(**a**) Flowchart for EM data processing and maps for color-coded local resolution estimation after processing with C1 and C4 symmetry, respectively.

(**b**) Representative size-exclusion chromatography profile on a Superose-6 column and SDS-PAGE of purified Kv3.1a. Multiple bands are due to complex N-linked glycosylation at N220 and N229.

(**c**) A representative electron micrograph (out of 4043), illustrating particle distribution of Kv3.1a.

Scale bar: 50 nm.

(**d**) Representative 2-dimensional class averages from the electron micrographs. Scale bar: 10 nm.

(**e**) Angular distribution of particles included in the final reconstructions.

(**f**) FSC curves of the refined model for processing with different masks.

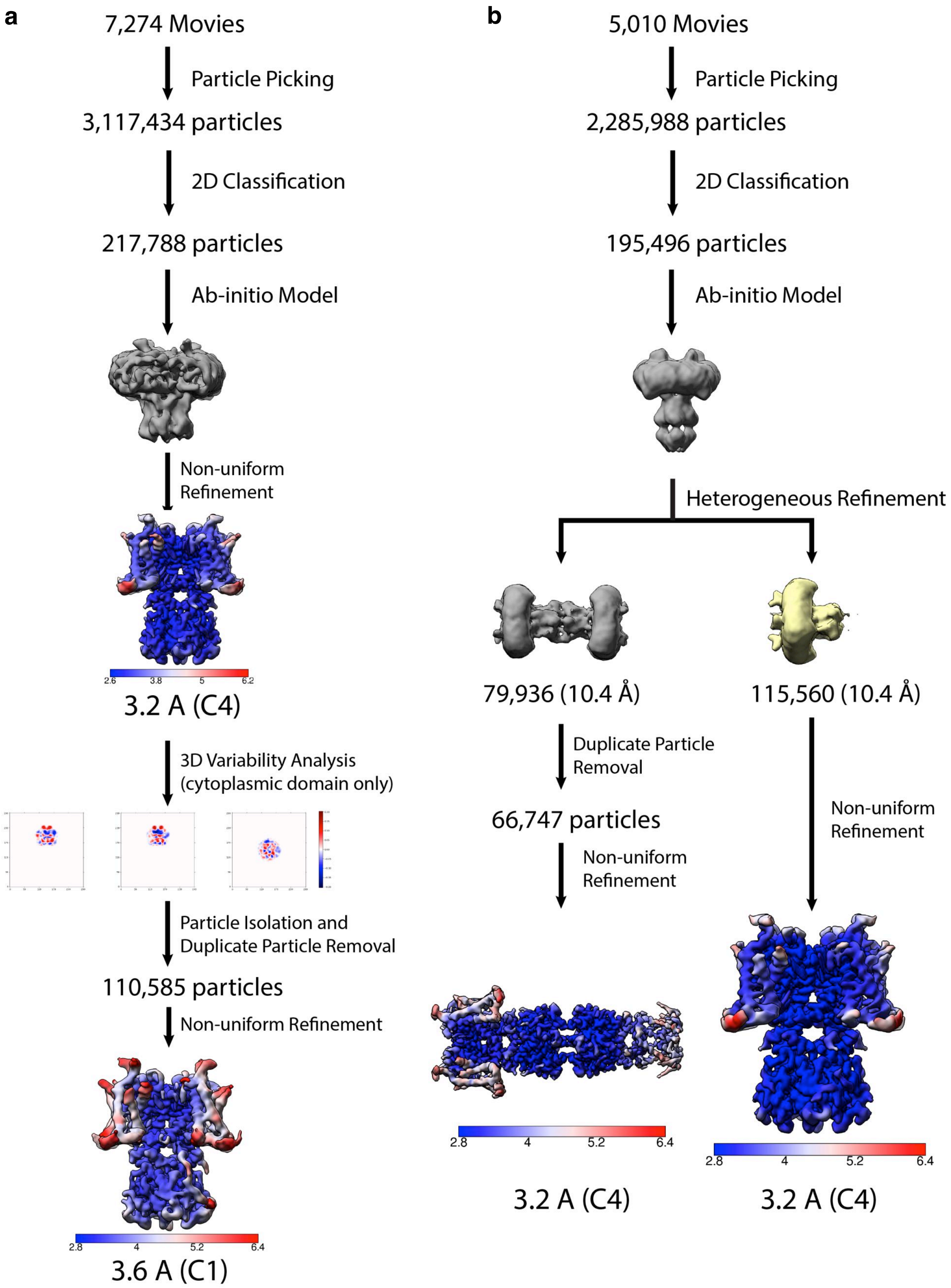

Supplemental Figure S3. Cryo-EM data processing of Kv3.1a in presence of EDTA or $ZnCl_2$.

(**a**) Flowchart for EM data processing including 3D variability analysis and maps for color-coded local resolution estimation after processing with C1 and C4 symmetry for a dataset obtained in presence of 1 mM EDTA.

(**b**) Flowchart for EM data processing and maps for color-coded local resolution estimation after processing with C4 symmetry for a dataset collected in presence of 400 µM $ZnCl_2$.

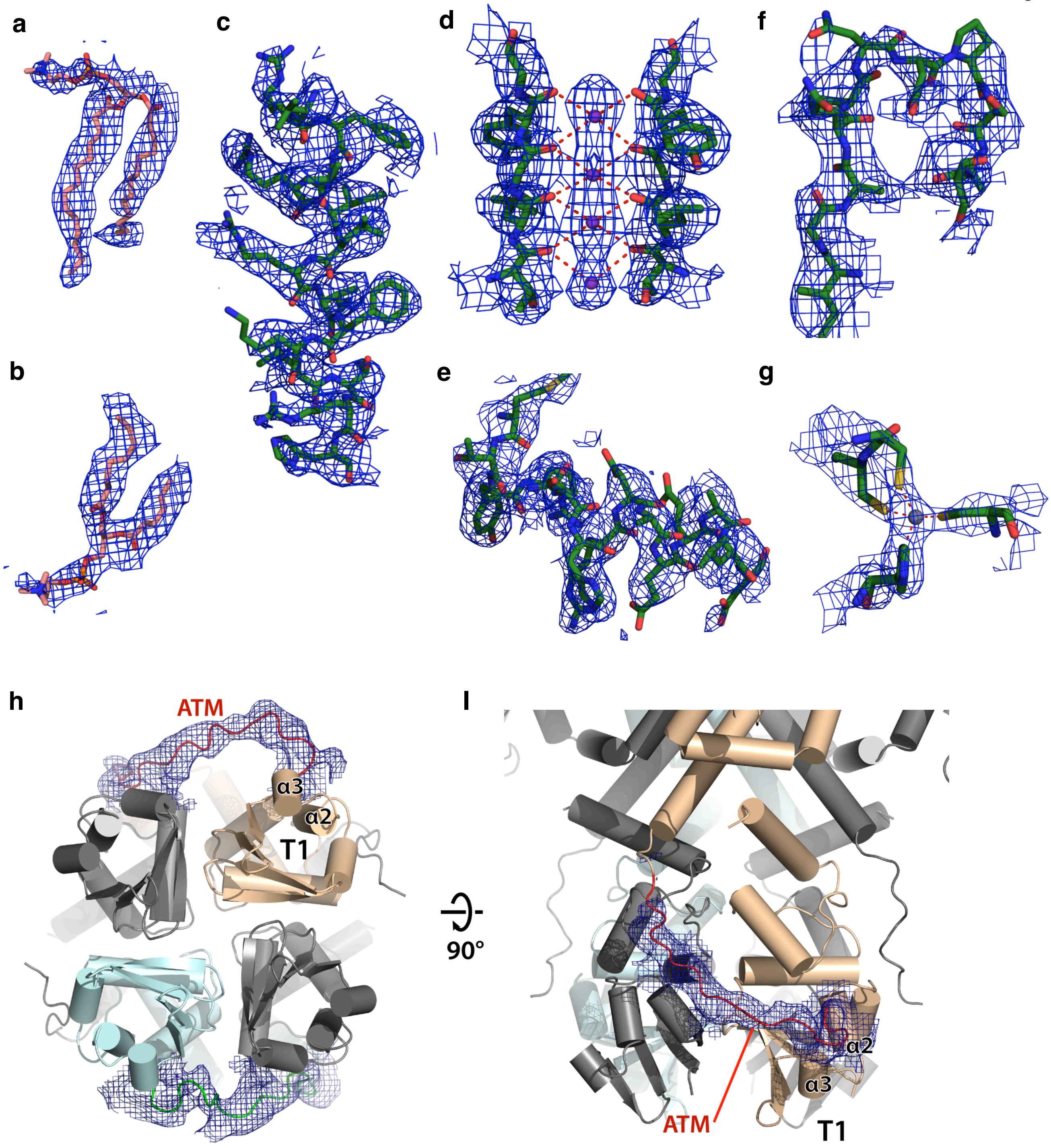

Supplemental Figure S4. EM density maps for representative segments in the Kv3.1a channel.
(**a**) EM density map for the lipid at site II (near PH helix/ turret)
(**b**) EM density map for the lipid at site I (near S4 and S4/S5L)
(**c**) EM density map for the S4 helix in the voltage-sensing domain (VSD)
(**d**) EM density map for the selectivity filter with coordinated K+ ions
(**e**) EM density maps for the α6 helix in the cytoplasmic T1 domain
(**f**) EM density maps for the turret domain
(**g**) EM density maps for the Zn^{2+} binding motif in the T1 domain
(**h-I**) EM density maps for the axonal targeting motif (ATM) and C-terminal extension for a particle subclass showing extra densities for two chains of the tetramer.

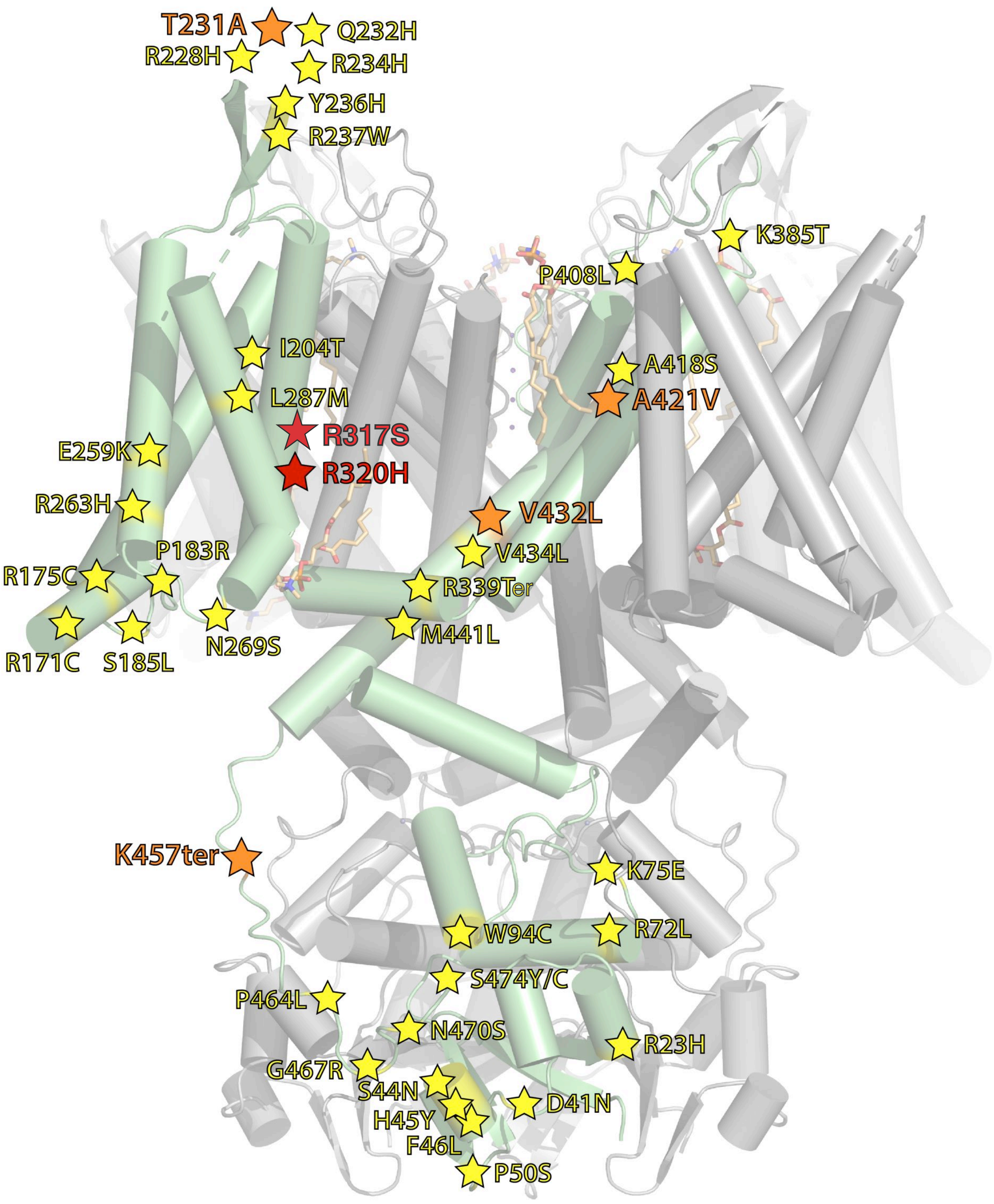

Supplemental Figure S5. Detailed mapping of disease mutations in the human *KCNC1* gene linked to EPM7 and related epileptic encephalopathies.

Variants of the human *KCNC1* gene associated with the autosomal dominant disorder EPM7 (progressive myoclonic eplilepsy-7) listed at www.malacards.org were mapped onto the structure of the Kv3.1a tetramer, represented as grey cartoon. A single protomer is coloured in light green to highlight mutations located at intersubunit interfaces. Red star symbols indicate mutations which have been characterized extensively in the literature and the epileptic phenotype of the variant has been confirmed. Mutations labeled with orange star symbols are annotated to be “likely pathogenic”. Yellow star symbols indicate amino acid positions of mutations with “uncertain significance” according to the variant database.

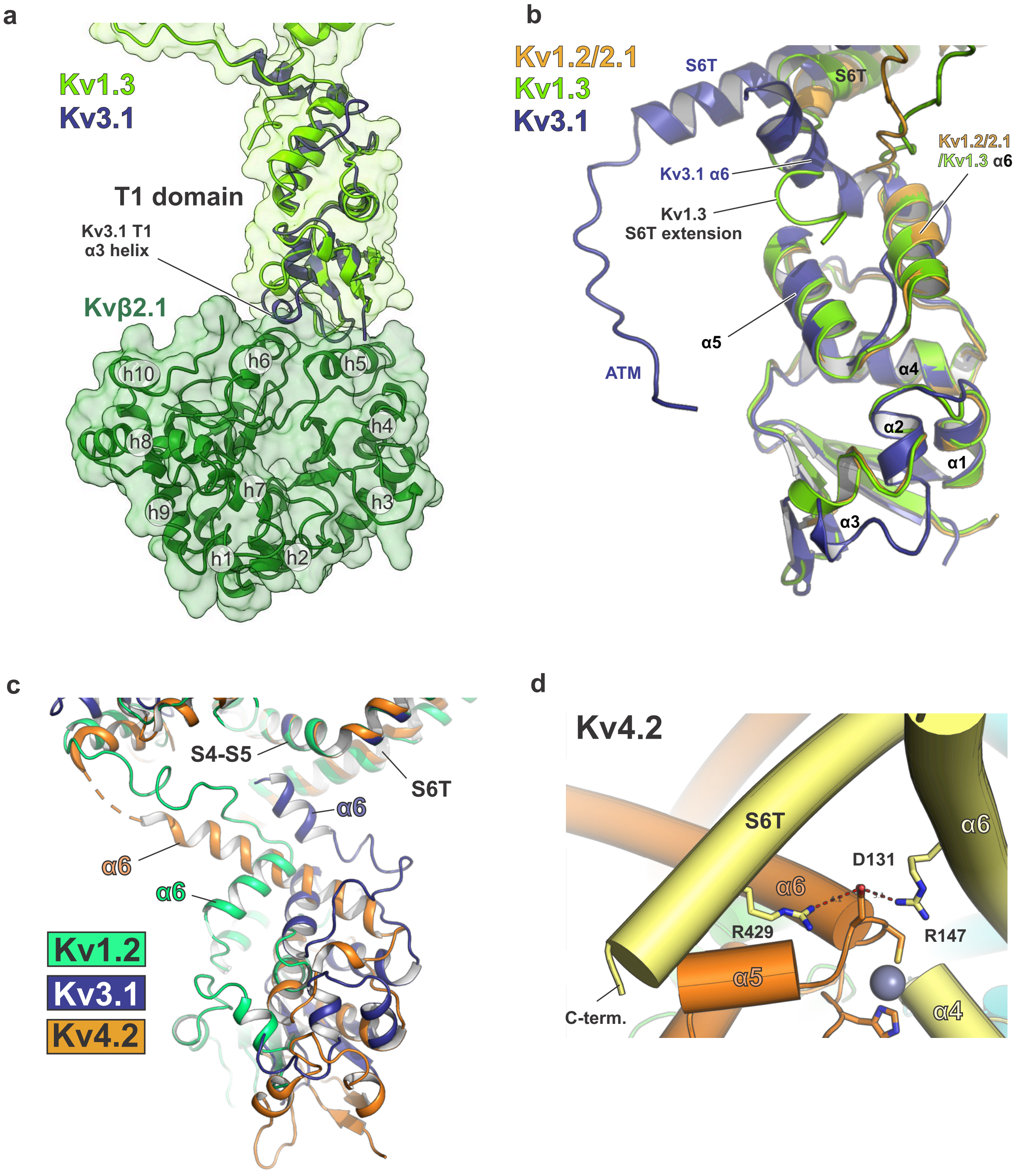

Supplemental Figure S6. Comparison of the T1 domain structure in Kv3.1a with T1 domains of representative members from the Kv1 and Kv4 subfamilies

(**a**) Superposition of the T1 domain from the Kv3.1a structure in the presence of Zn^{2+} (dark blue cartoon) with the T1 domain from the Kv1.3/β2.1 structure (green, pdb: 7EJI, shown as surface and cartoon), highlighting a clash of the Kv3.1 α3 helix with helix h6 of the β-subunit.

(**b**) Superposition of the T1 domain of Kv3.1a (dark blue) with the T1 domains of Kv1.2-2.1 (yellow, pdb: 6EBK) and Kv1.3 (green, pdb: 7EJ1) showing how S6T extensions in Kv1.3 would clash with an α6 helical arrangement similar to Kv3.1a.

(**c**) Comparison of the T1 arrangement in Kv3.1a (dark blue), Kv1.2-2.1 (green, pdb: 6EBK) and Kv4.2 (orange, pdb: 7F0J) after superposing the S4/S5 linker regions from one protomer of the respective full-length structures.

(**d**) Close contact between N–terminal T1 domain and c-terminal S6T segment in Kv4.2 (pdb 7F01). An inter-subunit salt bridge connects S6T with the cytoplasmic T1 domain. Neighboring chains are shown in orange and yellow, respectively.

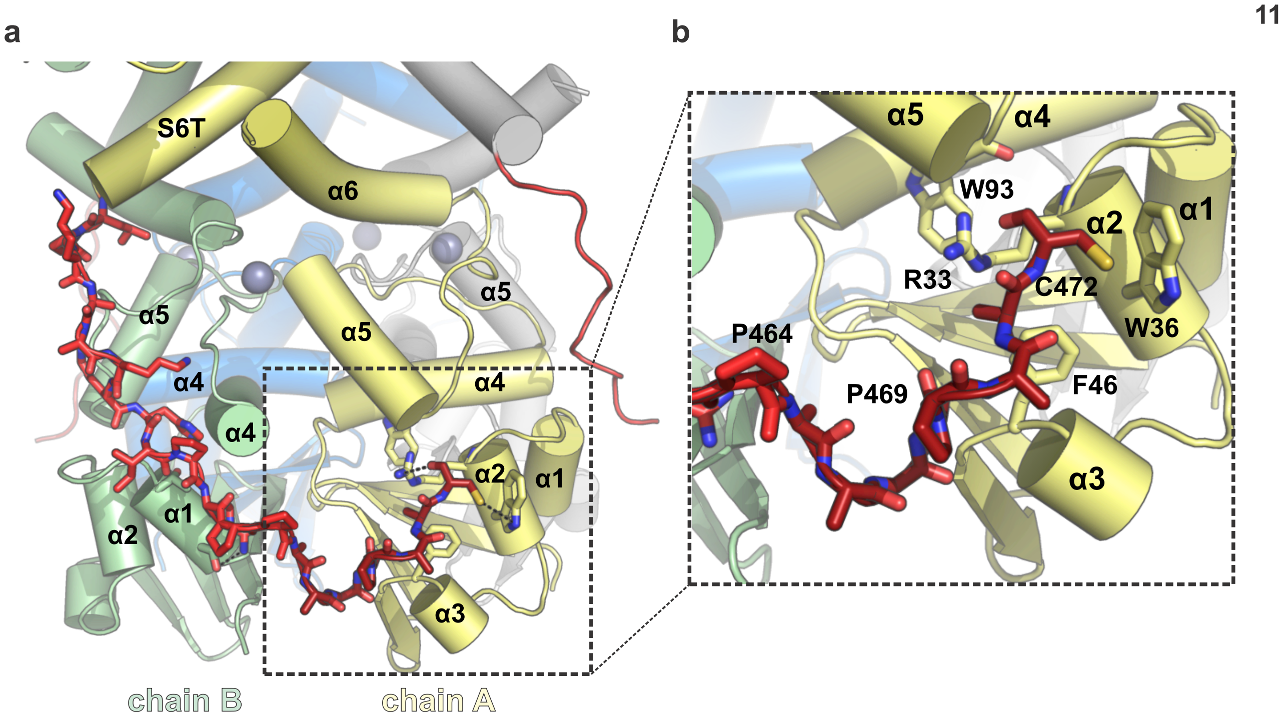

Supplemental Figure S7. C-terminal extension beyond the axonal targeting motif (ATM) interacts with two neighboring chains of the T1 domain.

(**a**) Close-up view of the T1 domain from the Kv3.1a with chains from different subunits shown as green, yellow, blue and grey cartoon. Residues 452-472 of the C-terminus from chain A are shown in stick representation. Residues 452-463 shown in bright red and residues 464-472 shown in dark red.

(**b**) Inset from A, showing intra-subunit interactions between residues of the C-terminal extension (residues 464-472, shown in dark red) and residues in the α2 and α3 helices (yellow cartoon) of the T1 domain.

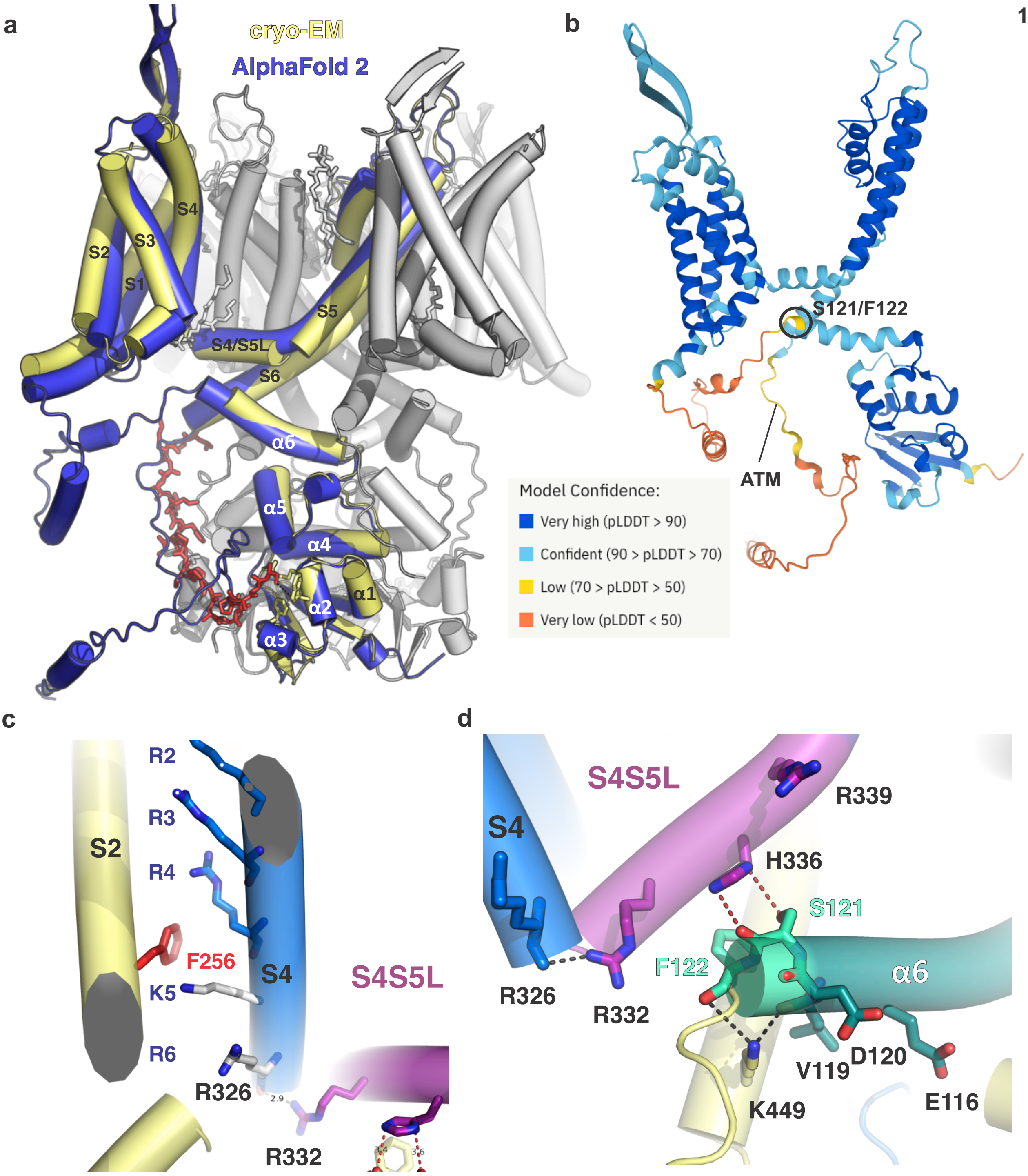

Supplemental Figure S8. Comparison of the Kv3.1a cryo-EM structure to model predicted by AlphaFold2 shows differences in the arrangement of S4 and the S4/S5 linker.

(**a**) Superposition of the protomer structure of human Kv3.1a predicted by AlphaFold2 (blue cartoon, taken from: alphafold.ebi.ac.uk/entry/P48547) onto 1 chain from the cryo-EM structure of Kv3.1a (yellow cartoon). The rest of the Kv3.1a tetramer is represented as grey cartoon.

(**b**) AlphaFold2 model for one protomer of Kv3.1a colored by level of confidence.

(**c**) Close-up showing the arrangement of the voltage-sensing S4 helix with positions of gating charges R2-R6 with respect to F356 of the CTC predicted by AlphaFold2. Compared to the experimentally determined cryo-EM structure, S4 is in a more upward-shifted position, with R4 located above F256 in the S1 segment.

(**d**) Close-up view of the S4/S5 linker region in the AlphaFold2 model, highlighting interactions to residues in S4 and α6 of the T1 domain. The model predicts a helical extension of two amino acids (S121 and F122) beyond the experimentally determined model. In this arrangement, the side chain of H336 interacts with the hydroxyl and backbone carbonyl groups of S121. K449 in S6T also interacts with a different set of residues (backbone carbonyls of V119 and G123). This moves the closest point of contact between S4/S5L and α6 in T1 closer to S4 (between R332 and H336), whereas this interdomain interaction is closer to S5 according to the cryo-EM structure (between H336 and R339, see main Figure 4 c). The R326/R332 H-bond between S4 and S4/S5 linker is present in the AI-predicted and the experimentally determined model.

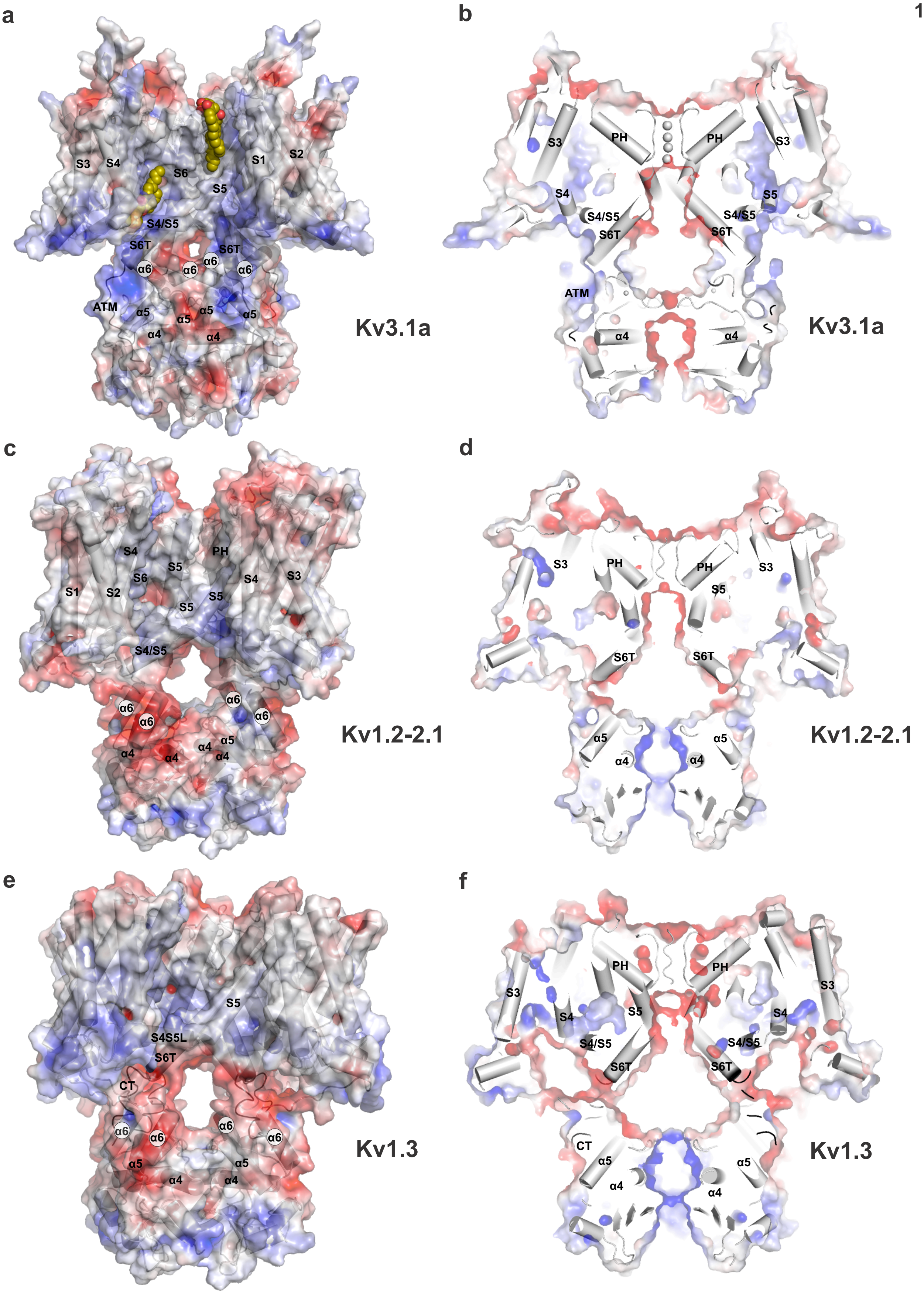

Supplemental Figure S9. Comparison of electrostatic surface potential distribution for human Kv channel structures of Kv3.1a, Kv1.2-2.1 and Kv1.3.

(**a**, **b**) Surface representation of human Kv3.1a in front view (A) and cross-sectional view (B).

(**c**, **d**) Surface representation of human Kv1.2-2.1 (pdb:6EBK) in front view (C) and cross-sectional view (D).

(**e**, **f**) Surface representation of human Kv1.3 in front view (E) and cross-sectional view (F).

Surfaces are coloured by electrostatic potential (red, $-5\ kT\ e^{-1}$; blue, $+5\ kT\ e^{-1}$).

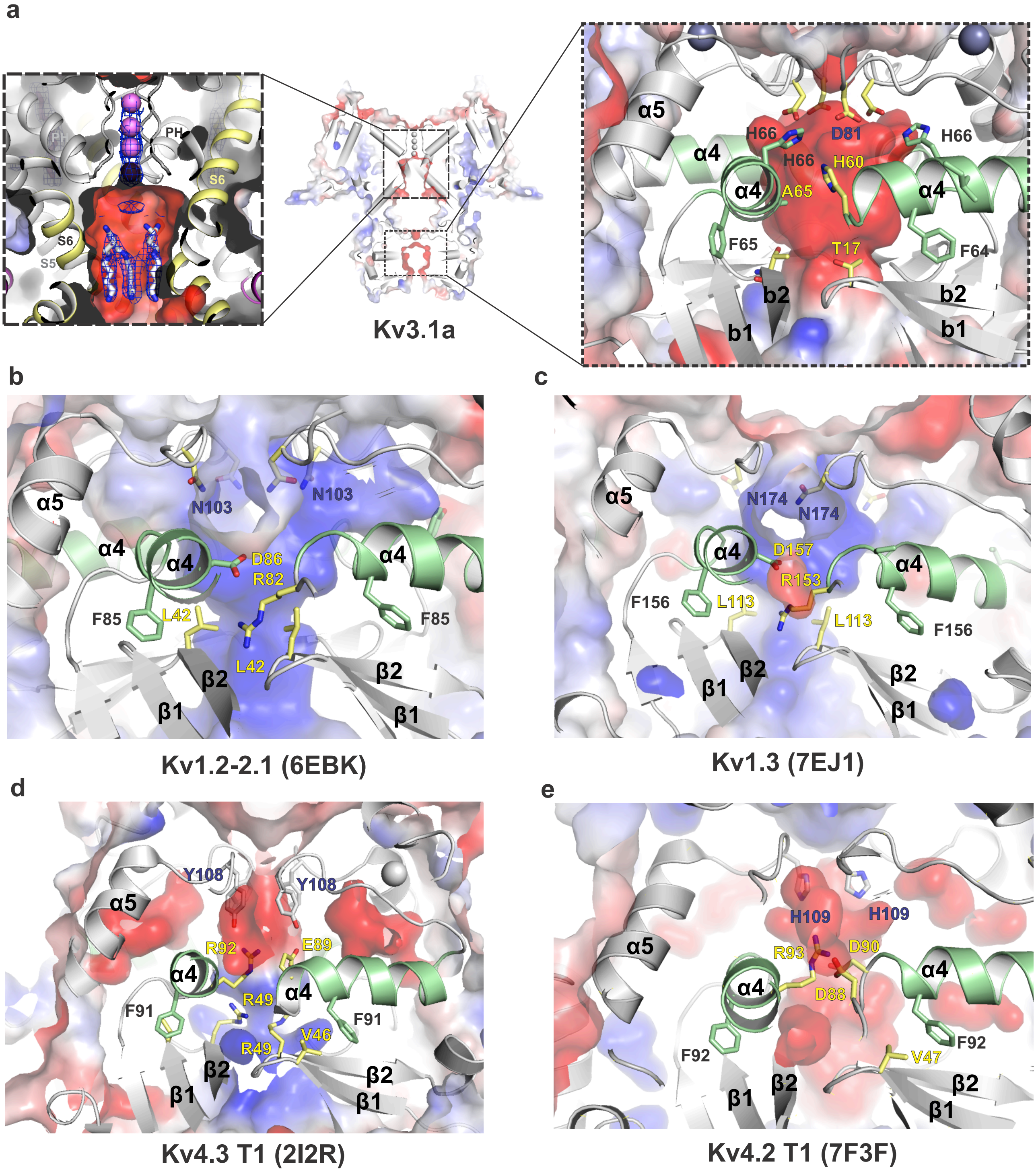

Supplemental Figure S10. Comparison of electrostatic surface charges near α4 in T1 of Kv3.1a and T1 domains from other representative channels of the Kv1 and Kv4 subfamilies.

(**a**) Slab view of the full length Kv3.1a structure in electrostatic surface representation. Left inset: Close-up view of the upper vestibule near the selectivity filter (SF). Densities for K^+ ions (purple spheres) in the SF and densities for unidentified small molecule(s) are shown as blue mesh.

Right inset: close-up view of the lower vestibule in T1 near helix α4, highlighting position of D81 in Kv3.1a.

(**b**) Close-up view of the lower vestibule in T1 near helix α4 in the full-length rat Kv1.2-2.1 structure (pdb 6EBK).

(**c**) Close-up view of the lower vestibule in T1 near helix α4 in the full-length human Kv1.3 structure (pdb 7EJ1).

(**d**) Close-up view of the lower vestibule near α4 for the rat Kv4.3 T1 structure (pdb 2I2R).

(**e**) Close-up view of the lower vestibule near α4 for the T1 domain in the full-length human Kv4.2 structure (pdb 7F3F).

Surfaces are coloured by electrostatic potential (red, −5 kT e^{-1}; blue,+5 kT e^{-1}). Residues in corresponding position to D81 in Kv3.1 are labeled in navy.

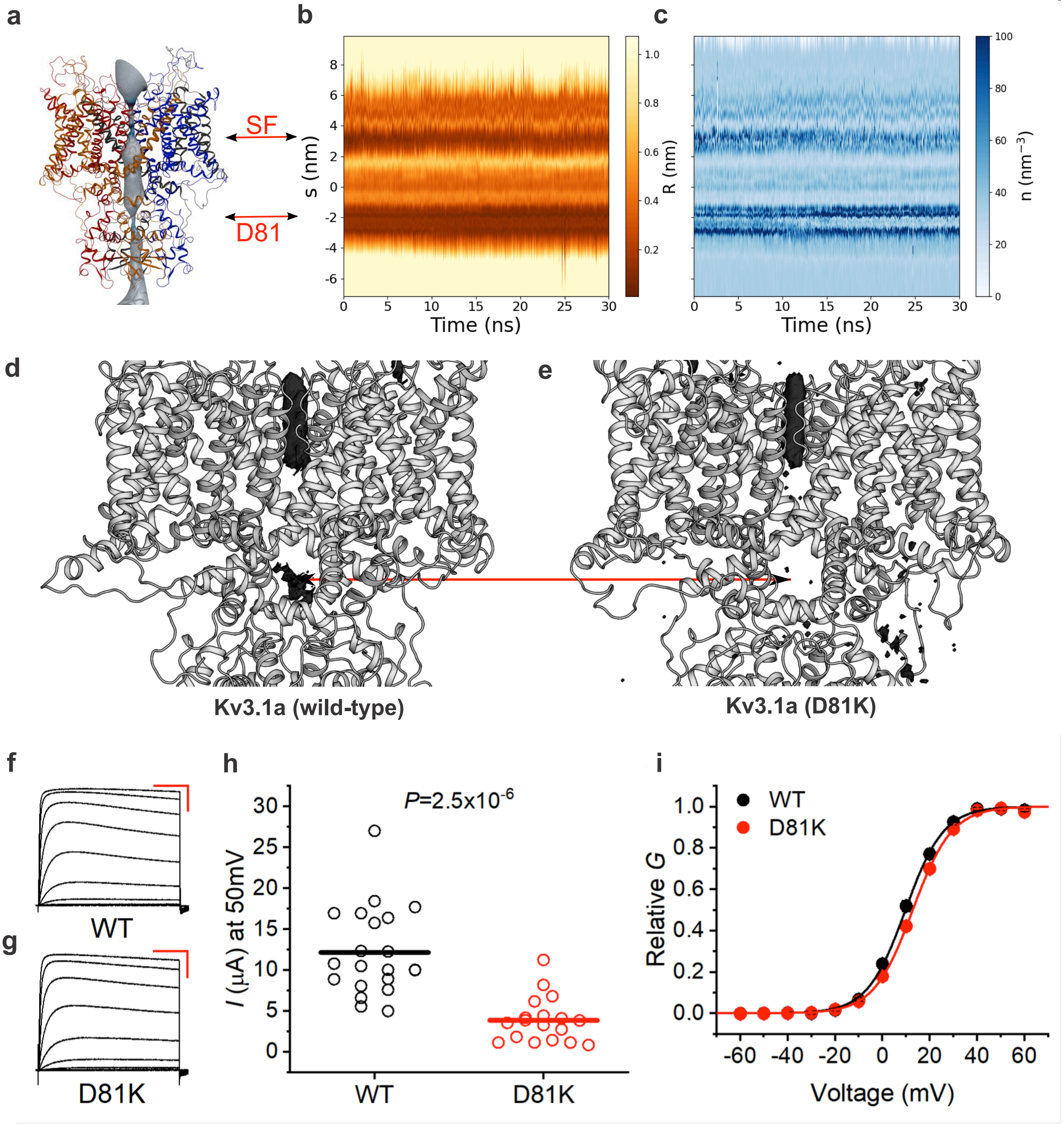

Supplemental Figure S11. Pore hydration properties and characterisation of the D81K mutant by MD simulations and TEVC recordings.

(a) Water density within the Kv3.1a channel calculated from unbiased MD simulations. The constriction site in the T1 domain at D81 is indicated by the red arrow symbol.

(b, c) Evolution of the pore radius in nm (B) and water density in nm^3 within the Kv3.1a pore (D) over simulation time (in nsec), showing that the pore region near the PVP hinge remains hydrated throughout the simulation.

(d, e) K^+ occupancy densities in simulations of wild-type (E) and D81K (F) mutant, illustrated at an isosurface value of 0.2 molecules/nm^3. The interstitial void occupancy by K^+ ions (indicated by the red arrow) is disrupted by the charge-inverting D81K mutation. The difference in abundance is seen in MD simulations at 0 mV and -300 mV.

(**f**, **g**) Current responses for wildtype and D81K evoked by 400-ms step depolarizations from a holding voltage of −100 mV. The steps are delivered in increments of 10 mV from -60 mV to 50 mV and the start-to-start interval is 5 s. The horizontal and vertical scale bars indicate 100 ms and 1 μA, respectively.

(**h**) Representative current responses from D81K and WT Kv3.1a expressing oocytes (at + 50 mV, circles) and mean value (bars) for WT (black) and D81K mutant (red). P value for one-sided ANOVA test is indicated on the graph. Data are averages from 21 (WT) and 18 (D81K) oocytes, respectively.

(**I**) G-V curves for wildtype Kv3.1a (black circles) and mutant D81K (red circles). Data are averages from 9 oocytes, respectively. Solid lines represent the best first-order Boltzmann fits.

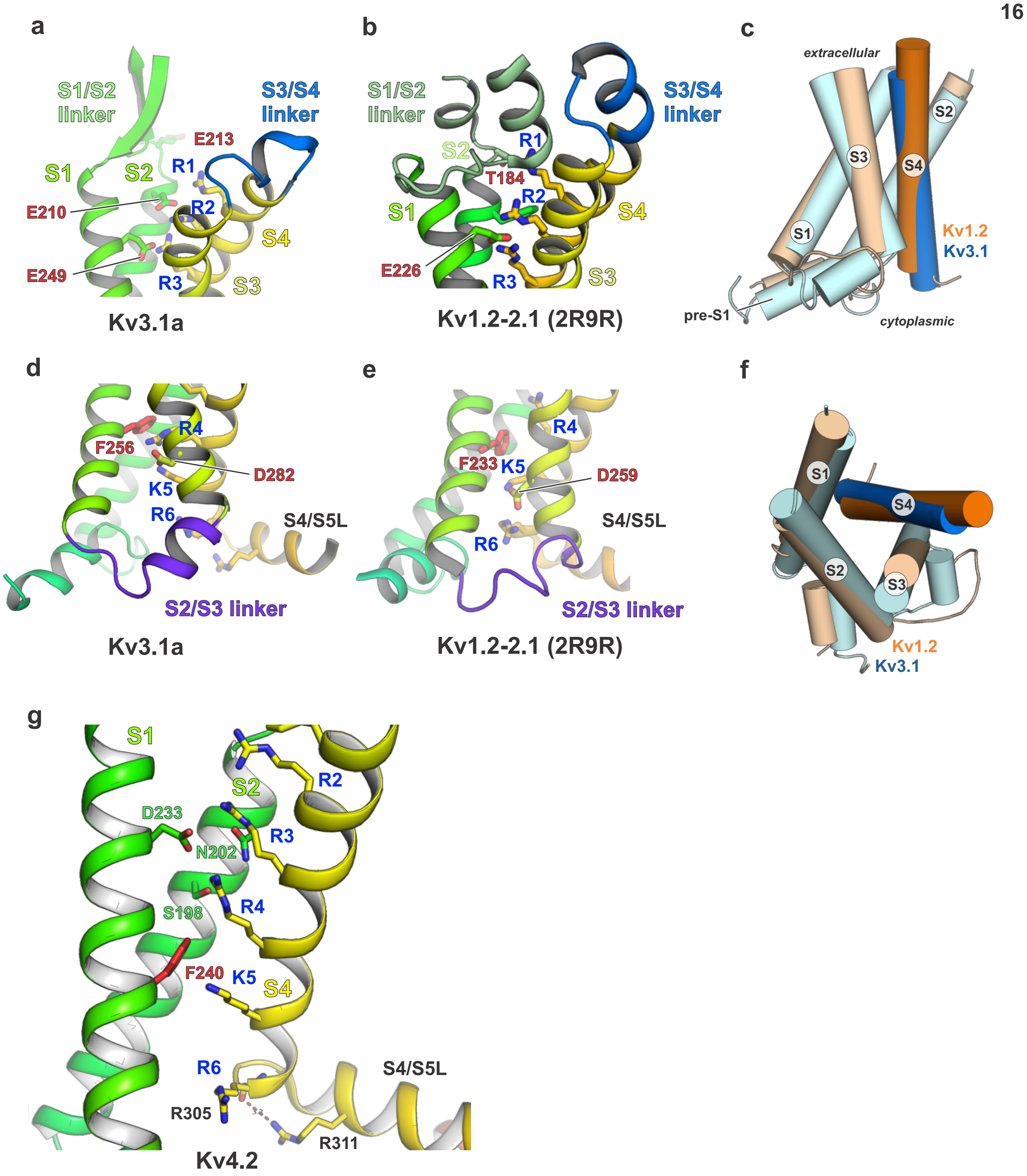

Supplemental Figure S12. Comparison of extra- and intracellular linker arrangement in the voltage-sensing domains of Kv3.1a and Kv1.2-2.1 and VSD arrangement in Kv4.2.

(**a**) Cartoon representation of the extracellular half of the Kv3.1a VSD (S1-S4) with S1/S2 and S3/S4 linkers shown in green and blue cartoon representation.

(**b**) Same region as in A but for the Kv1.2-2.1 VSD (pdb: 2R9R).

(**c**) Superposition of the VSDs from Kv3.1a (blue) and Kv1.2 (orange), using S1-S3 as a reference point. S4 (shown in a bolder colour than S1-S3) is upward shifted for Kv3.1 by one helical turn, compared to the position of S4 in Kv1.2.

(**d**) Cartoon representation of the intracellular half of the VSD (S1-S4) in KV1.3a, with the S2/S3 linkers shown in purple cartoon representation.

(**e**) Same as in D, but for the respective S2/S3 linker in Kv1.2-2.1 (pdb: 2R9R).

(**f**) Extracellular view of the VSD superposition of Kv3.1 and Kv1.2 in C.

(**g**) VSD arrangement in Kv4.2 (pdb: 7F0J).S3 is ommited for clarity. A salt bridge between R6 and R311 in S4/S5L is indicated by dashed lines.

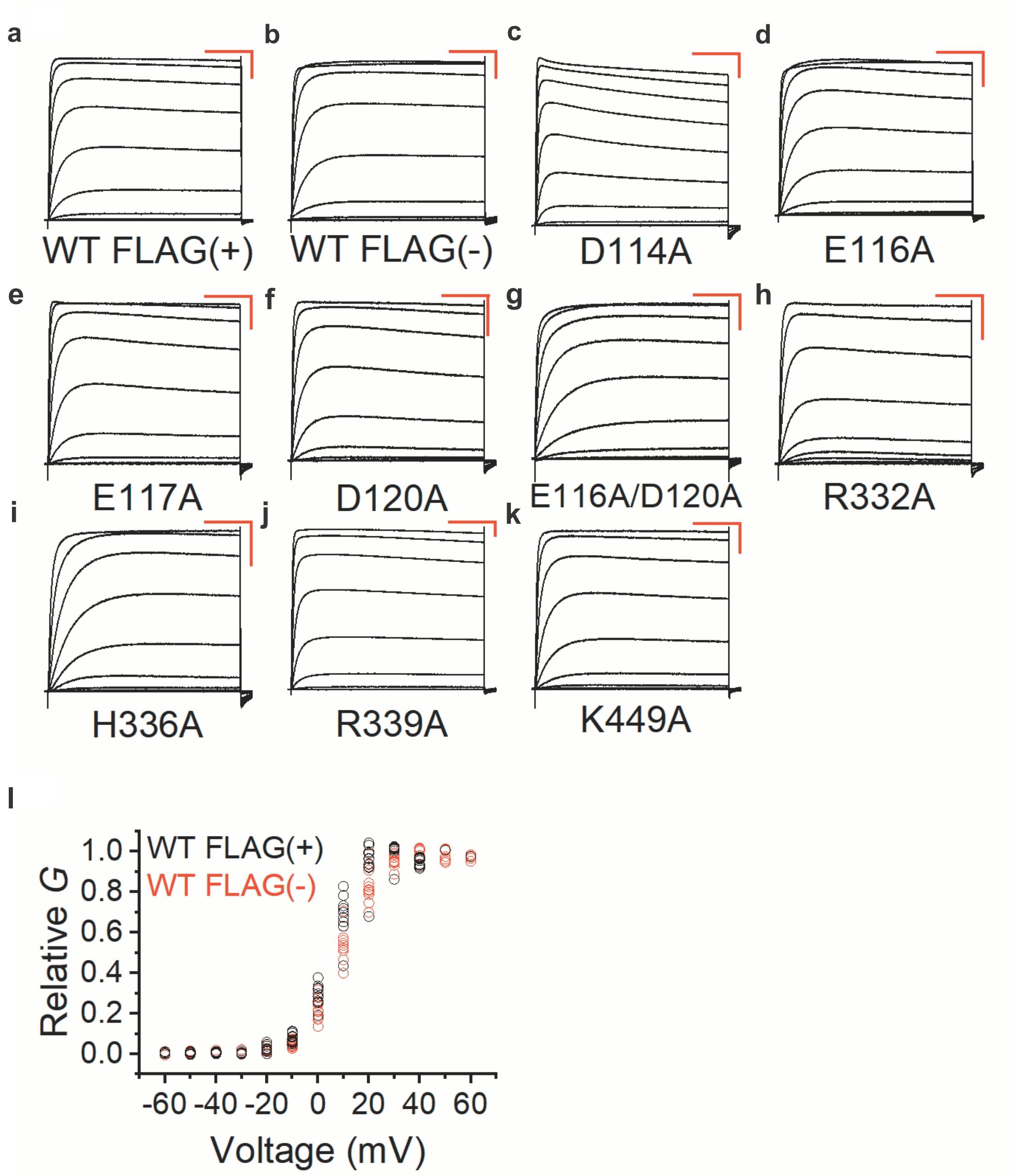

Supplemental Figure S13. TEVC recordings of Kv3.1a channels in *Xenopus* oocytes.

(a-b) Representative whole-oocyte currents of wild type Kv3.1a (WT) with (+) and without (-) C-terminal FLAG tag. Currents are evoked by 400-ms step depolarizations from a holding voltage of −100 mV. The steps are delivered in increments of 10 mV from -60 mV to 50 mV, and the start-to-start interval is 5 s. The horizontal and vertical scale bars indicate 100 ms and 1 μA, respectively.

(**c-k**) Representative whole-oocyte currents of several alanine replacement mutants introduced into the wild type Kv3.1a construct. The voltage protocol and scale bars are as described for panel a. For R332A and K449A, steps are extended to + 60 mV.

(**l**) G-V curves for wildtype Kv3.1a with (+) and without (-) the C-terminal FLAG tag. Data are scatter plots from 11 and 10 oocytes, respectively.

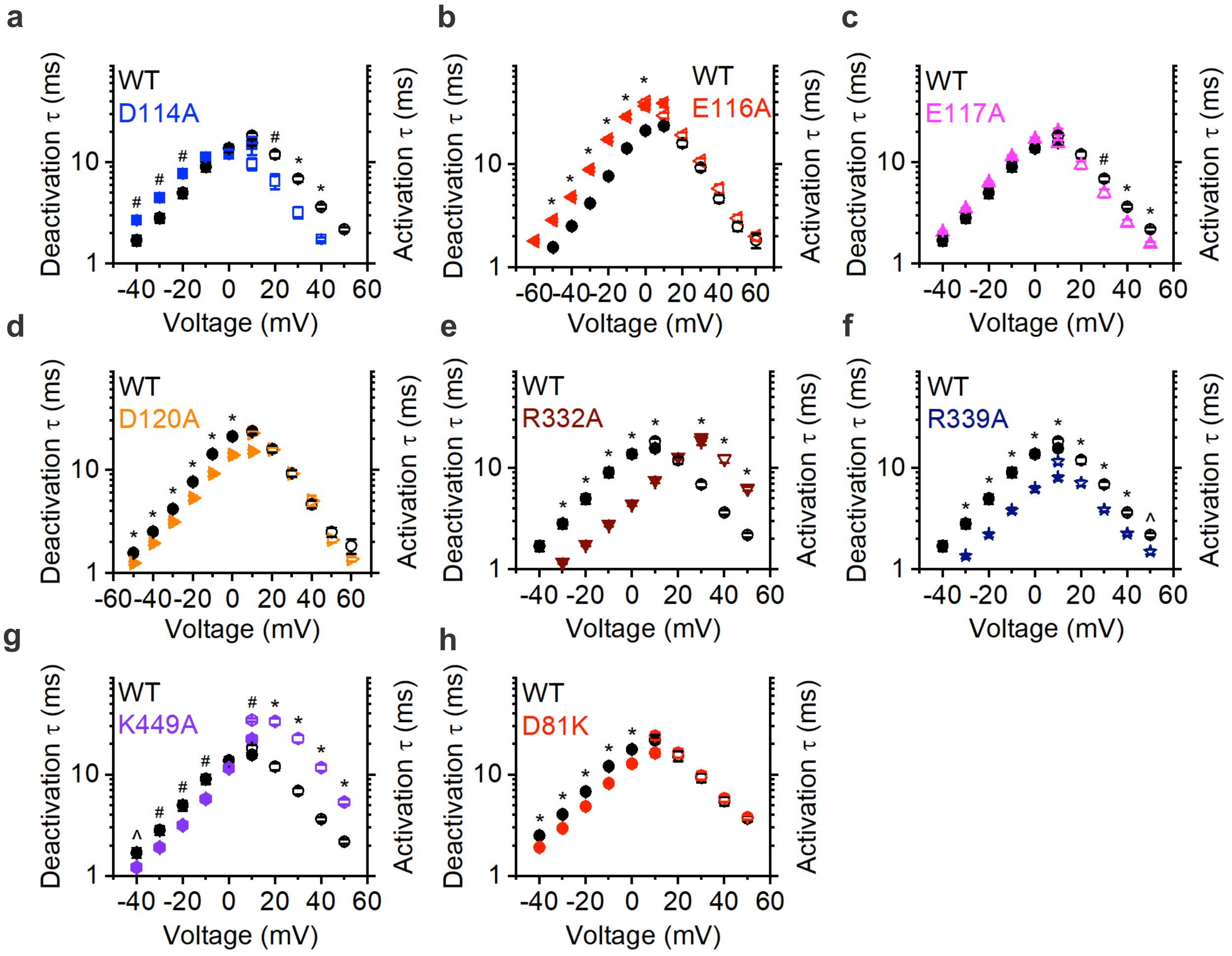

Supplemental Figure S14. Activation and deactivation time constants for mutant Kv3.1a channels in comparison to wildtype Kv3.1a.

(a) The voltage dependence of the time constants of activation (hollow symbols) and deactivation (filled symbols) from wildtype Kv3.1a (black circles) and mutant D114A (blue circles). Symbols and error bars represent the mean ± SEM. Error bars are smaller than the symbol size.

(b-h) same as (**a**), but data for mutants E116A, E117A, D120A, R332A, R339A, K449A and D81K respectively.

In each group, n=8-10 oocytes. To evaluate differences relative to WT, we use the one-way (or Kruskal-Wallis if necessary) ANOVA test. ^ $P<0.05$ # $P<0.01$ * $P<0.001$.

Exact values for n and P for the time constant comparisons at each voltage are listed in the source file included with this study.

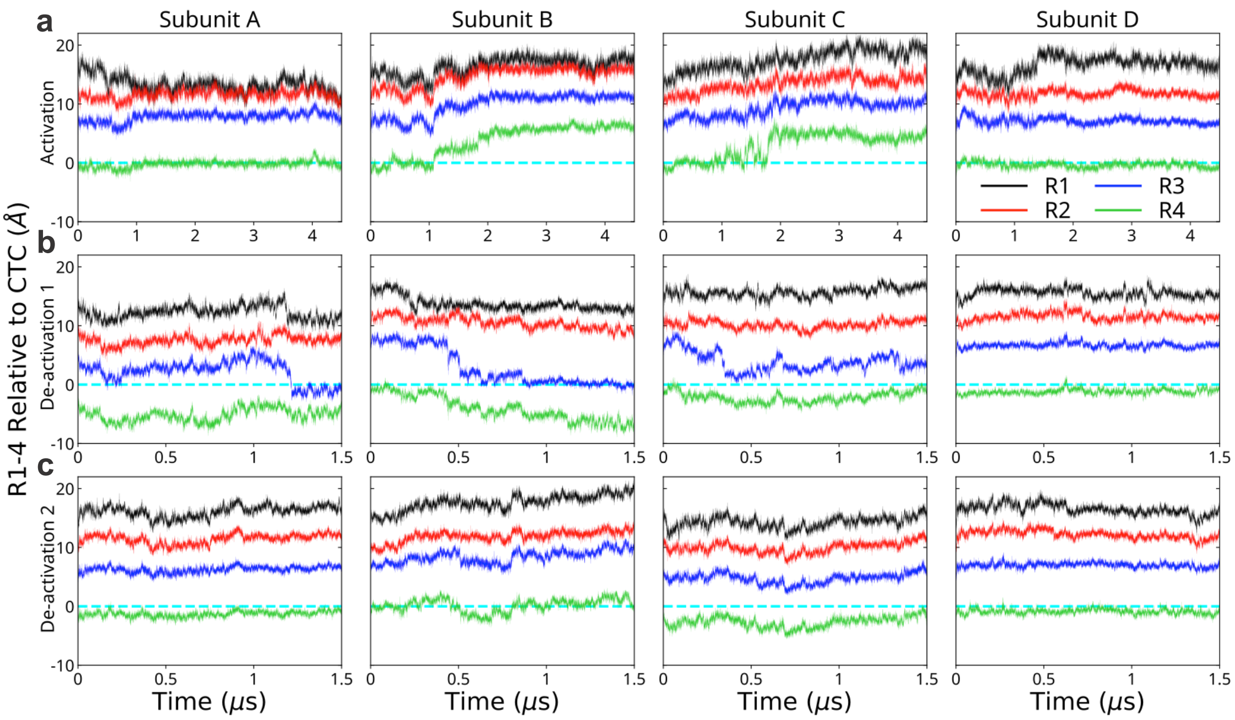

Supplemental Figure S15. Z-position of the S4 gating charges R1-R4 relative to the CTC in Kv3.1a for subunits A-D in response to activating and deactivating potentials.

(a) Z-position of the S4 gating charges R1-R4 within the Kv3.1a subunits relative to the CTC (cyan dashed line) in response to application of activating (+300 mV) potentials.

(b-c) Z-position of the S4 gating charges R1-R4 within the Kv3.1a subunits relative to the CTC (cyan dashed line) in response to deactivating (-650 mV) potentials.

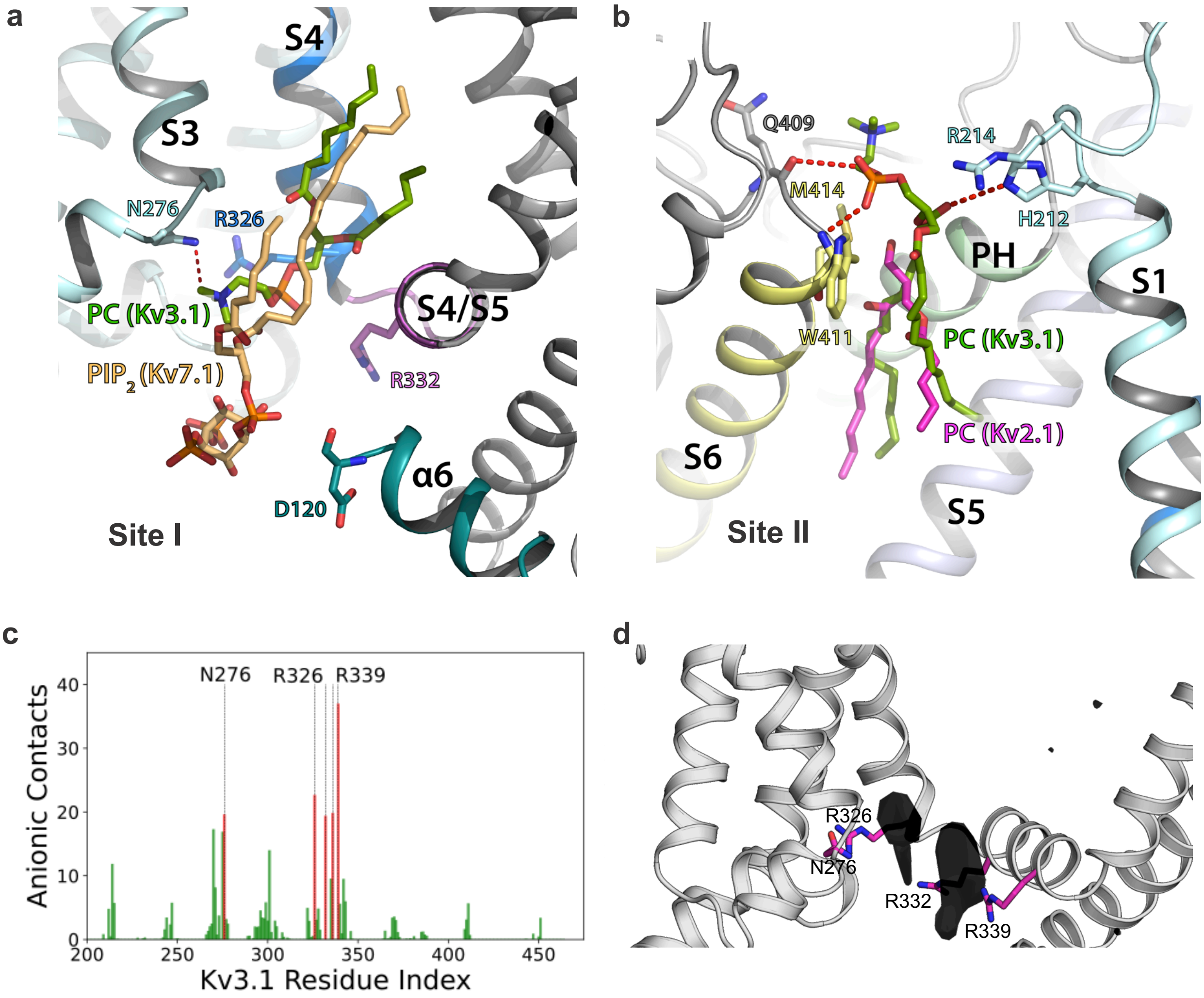

Supplemental Figure S16. Analysis of lipid/protein interactions in the human Kv3.1a channel.

(**a**) Lipid-protein interactions at Site I: a phospholipid (PC, green sticks) is bound near the S4/S5 linker (purple) and voltage-sensing helix S4 (blue) of Kv3.1a. The binding site exhibits similarity to the known PIP$_2$ (wheat-coloured sticks) binding site in the structure of Kv7.1 (pdb: 6V01).

(**b**) Lipid-protein interactions at Site II: a phospholipid (PC, green sticks) is bound at the interface between S6 (yellow) and PH (green) in Kv3.1a compared to phosphatidylcholine (PC, pink sticks) in the Kv1.2/Kv2.1 structure (pdb: 4JTC).

(**c**, **d**) MD simulations demonstrate that anionic lipids predominate interactions at lipid site I. In contrast, no lipid preferentially localizes to site II.

(**c**) Normalized number of contacts between Kv3.1a residues and headgroups of PS/PIP$_2$. A contact was assumed if a residue's bead was within 5.5 Å of the lipid headgroup's bead and contacts were subsequently averaged across the four subunits. Residues with a contact frequency > 17.5% (half the ratio of anionic lipids in the inner leaflet) are coloured red.

(**d**) Densities of the anionic lipids calculated from CG simulations illustrated at one of the subunit interfaces at an isosurface value of 4.0 molecules/nm^3.

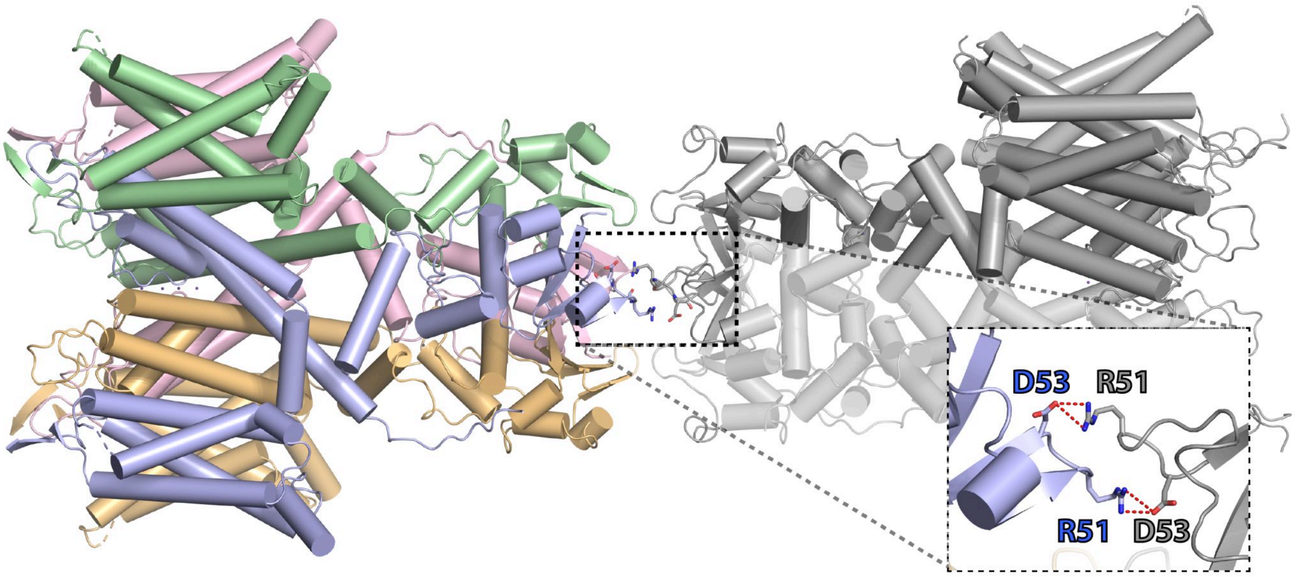

Supplemental Figure S17. Illustration of dimer interface interactions for a subset of particles forming Kv3.1a dimers in the dataset with 400 µM $ZnCl_2$.

Cartoon representation of the Kv3.1a dimer arrangement observed for a subset of particles present in the $ZnCl_2$-containing EM dataset.

Inset: Close-up showing that the dimer interaction is facilitated by stabilizing salt bridges between the side chains of D51 and R52 in the cytoplasmic T1 domain.

	Region	*n*	$V_{0.5}$ (mV)	*P*	*z* (e_0)	*P*
WT FLAG(+)		29	8.5 ± 0.6		3.7 ± 0.1	
WT FLAG(-)		11	8.9 ± 0.7		3.8 ± 0.2	
D81K	Alpha 4/alpha5 linker	9	12.7 ± 0.6	0.014	3.1 ± 0.1	0.493
D114A	Alpha 6	9	-1.9 ± 0.9	3.0×10^{-6}	3.8 ± 0.1	0.640
E116A	Alpha 6	10	4.4 ± 0.8	6.0×10^{-3}	3.2 ± 0.1	0.068
E117A	Alpha 6	10	6.4 ± 0.9	0.274	3.6 ± 0.2	0.437
D120A	Alpha 6	11	11.0 ± 1.6	0.090	3.6 ± 0.3	0.833
E116A/D120A	Alpha 6	10	5.1 ± 0.8	0.027	3.0 ± 0.2	8.3×10^{-3}
R332A	S4-S5	10	27.9 ± 1.7	1.1×10^{-10}	2.9 ± 0.2	2.7×10^{-3}
H336A	S4-S5	9	10.3 ± 1.1	0.260	3.8 ± 0.1	0.941
R339A	S4-S5	11	12.6 ± 1.2	0.017	3.7 ± 0.1	0.378
K449A	S6	10	21.4 ± 1.0	2.7×10^{-8}	3.6 ± 0.1	0.409

Supplemental Table 2. Summary of best-fit Boltzmann function parameters of G-V curves obtained for wildtype or mutant Kv3.1a channels.

Parameters are expressed as mean ± SEM from n oocytes. WT values are global.

P values are compared to WT from the same batch or consecutive combined batches. One-way ANOVA or Kruskall-Wallis ANOVA.

Supplementary Results & Discussion

Kv3.1a/Zn^{2+}- structure does not support evidence for secondary Zn^{2+}-binding sites

Gu *et al*. report that zinc reversibly binds to Kv3.1 at several sites other than T1 domain's zinc finger and this has various effects on ion channel activity, depending on where the sites are located [1]. Additionally, our structures suggest another pocket resembling zinc finger motif formed by H60, H66 and D81. Therefore, we collected datasets of Kv3.1 in the presence of 400 µM $ZnCl_2$ or 1 mM EDTA to resolve any extra zinc-binding sites which may exist in Kv3.1a. However, no conformational change of Kv3.1a was observed between the two maps and no inorganic ion was seen in the $ZnCl_2$ sample in the four suspected sites (H60/D81, C208/H212, H381/H383 and H459). The first hypothesis to explain this is that zinc ions do not bind to Kv3.1 other than the zinc finger motif in the near-open state. There is also the possibility that zinc ions do not bind tightly to the suspected sites.

While we did not find evidence for additional zinc-binding sites with our structural biology efforts, we serendipitously discovered a potential cause of higher-order dimerisation seen with some tetrameric ion channels (Figure S3 b). Ion channels with T1 domain often show dimerisation at the distal end of T1 domain, for example a plant hyperpolarisation-activated K^+ channel KAT1 [2]. On visual examination of electron micrographs, we found that Kv3.1a shows increased dimerisation in the presence of $ZnCl_2$ but does not form a dimer with 1 mM EDTA. Reconstruction of the dimer showed that this oligomer was stabilised by R51 and D53 of one subunit interacting with D53 and R51 with the other subunit, respectively (Figure S17). It is beyond the scope of this study to investigate the mechanism of such oligomerisation and its physiological significance, however our observation provides basis for further exploration of such feature.

Subclass map of Kv3.1a structure in EDTA shows C-terminus encircling T1 domain

Kv3.1a cryo-EM maps show protein density on the lateral side of T1 domain at low contour levels. In order to better resolve this region, we performed 3D variability analysis on Cryosparc with mask around the cytoplasmic domain. One of the modes showed this feature more clearly for two subunits diagonal to each other, and it was identified as further C-terminal extension of axonal targeting motif (ATM).

In this extended state, ATM makes contact with two T1 domains, the one of neighbouring subunit on its N-terminal side and T1 of its own subunit on the C-terminal side. If extrapolated to all four

subunits, this would create a "ring" of ATM strand around the lateral side of T1 domain. Such positioning of ATM is similar to that of KChIPs, which bind to the sides of T1 domains of Kv4 channels [3, 4]. This suggests ATM's involvement in coordinating Kv3.1a gating between T1 domain and pore domain. This is supported by H459A mutation leading to shift in Kv3.1 activation threshold without affecting the channel's sensitivity to zinc [1], as well as the channel-inactivating K457Δ truncation associated with a rare form of epilepsy.

Small molecules occupy the aqueous cavity below Kv3.1 selectivity filter

We observe non-peptide density in the aqueous cavity on the cytoplasmic side of selectivity filter of Kv3.1a. Although the shape of the density is probably distorted due to its location on the four-fold symmetry axis, the generally tubular shape of this density parallel to the symmetry axis appears to be a genuine feature of this small molecule. This density fits well with the shape and size of polyamines such as spermidine coordinating with water (Figure. S10 a), as well as short-chain phospholipids and free fatty acids. Similar densities are also present in the cytoplasmic cavities of Kv1.2/2.1 chimera [5], NavAb [6], TRAAK [7], KirBac3.1 [8] and Kv4.2 [9] channels, all of them in the open state, suggesting that the presence of small molecules at this site is a common feature for ion channels.

Some features of this aqueous cavity suggests this molecule being a polyamine. First, the surface of this cavity is strongly negatively charged (Figure S9 b), an ideal environment for occupation by positively charged molecules. Although there is no report of Kv3.1 (or any Shaker superfamily channel) being modulated by polyamines, they are known open-pore blockers of inward-rectifying potassium channels (such as KirBac3.1) by occupying analogous site [8]. Interestingly, all known small molecule pore-blockers of Kv3.1 such as tetraethylammonium (TEA), 4-aminopyridine (4-AP), 3,4-diaminopyridine (3,4-DAP), and dimethylguanidine (DMG) feature amine group and are thought to occupy this aqueous cavity. These provide circumstantial evidence for this molecule being an amine derivative.

There is also the possibility of this molecule being a phospholipid or free fatty acid. While there is little structural evidence of this unlike the polyamine hypothesis, functional data exist for the modulation of Shaker superfamily channels by free fatty acids. For example, arachidonic acid is known to inhibit Kv4.2 and Kv1.4 [10]. It is also a rationally most sensible explanation as the aqueous cavity is still located within the transmembrane domain and phospholipids have been observed partially occupying this cavity in Nav1.4, albeit with a significantly different binding mode [11]. It is also possible that a detergent is trapped in the open pore, similar to the configuration observed in

the Nav1.4 structure from electric eel [12]. In contrast to the Nav1.4 structure where the pseudo 4-fold symmetry is broken due to the complex formation with the β1-subunit and the shape of the density matches well with a digitonin molecule, the 4-fold symmetrical appearance of the densities below the selectivity filter in our Kv3.1a structure could be an EM processing artefact. Because of its location on the 4-fold axis of symmetry, the density from the bound molecule is possibly distorted and may not faithfully resemble the shape of a detergent/lipid/free fatty acid, even if the data is processed in C1.

We performed metabolomics mass spectrometry in an effort to determine the identity of this molecule (data not shown), however the result was inconclusive. We did not detect presence of polyamines or other unique small molecules, however this might have been due to the molecules still being trapped in the protein as it collapsed in the denaturation process. We detected a number of fatty acids, most notably eicosanoic acid, however they might have been products of the fragmentation of annular phospholipids. Therefore, we could not ascertain the identity of this molecule and therefore excluded it from structural model despite its potential importance in ion channel function.

Supplementary Methods

Coarse Grain Simulations

Unresolved residues in the cryo-EM structure within the S1/S2, S3/S4 and T1/S1 loops were built using Modeller [13]. The long unresolved loop between the T1 α6 and S1 helices was modelled as a combination of two unstructured regions $_{121}$SFGGAP$_{126}$ and $_{159}$DSPDGRPGGF$_{168.}$ Coarse-grained simulations were performed using the Martini 2.2 force field [14] with the protein embedded in an asymmetric model membrane [15] using the Martini Bilayer Maker [16] of CHARMM-GUI [17]. After energy minimization and equilibration for 20 ns, three replicates of the system were simulated for 20 µs each using Gromacs 2020 [18] with the protein backbone beads restrained to allow convergence of lipid interactions. The final 7.5 µs of the simulation trajectories were used for analysis using the PyLipID [19] and MDTraj [20] libraries . A contact was assumed if a residue's bead was within 5.5 Å of a lipid head-group bead and occupancy probability density calculations were performed using a grid of 2 Å resolution.

Free-Energy perturbation calculations following the methodology of Corey et al. [21] were performed to ascertain the identity of lipid site I without preferential interactions in the CG

simulations. The PC lipid in CG resolution was alchemically transformed into an anionic PA lipid previously identified to occupy the analogous site in prokaryotic KcsA channels [22, 23]. Coulomb and van-der-Waals interactions were perturbed separately using 10 windows each along the λ chemical space. Each λ window was energy-minimized, equilibrated and then simulated for 300 ns using a leapfrog stochastic dynamics integrator. The *alchemlyb* library [24] was used to calculate energies from the individual windows through the BAR method [25]. Results were calculated as a mean of three independent perturbation simulations.

References

1. Gu, Y., J. Barry, and C. Gu, *Kv3 channel assembly, trafficking and activity are regulated by zinc through different binding sites.* J Physiol, 2013. **591**(10): p. 2491-507.
2. Clark, M.D., et al., *Electromechanical coupling in the hyperpolarization-activated K.* Nature, 2020. **583**(7814): p. 145-149.
3. Wang, H., et al., *Structural basis for modulation of Kv4 K+ channels by auxiliary KChIP subunits.* Nat Neurosci, 2007. **10**(1): p. 32-9.
4. Pioletti, M., et al., *Three-dimensional structure of the KChIP1-Kv4.3 T1 complex reveals a cross-shaped octamer.* Nat Struct Mol Biol, 2006. **13**(11): p. 987-95.
5. Matthies, D., et al., *Single-particle cryo-EM structure of a voltage-activated potassium channel in lipid nanodiscs.* Elife, 2018. **7**.
6. Wisedchaisri, G., et al., *Resting-State Structure and Gating Mechanism of a Voltage-Gated Sodium Channel.* Cell, 2019. **178**(4): p. 993-1003 e12.
7. Brohawn, S.G., E.B. Campbell, and R. MacKinnon, *Physical mechanism for gating and mechanosensitivity of the human TRAAK K+ channel.* Nature, 2014. **516**(7529): p. 126-30.
8. Clarke, O.B., et al., *Domain reorientation and rotation of an intracellular assembly regulate conduction in Kir potassium channels.* Cell, 2010. **141**(6): p. 1018-29.
9. Kise, Y., et al., *Structural basis of gating modulation of Kv4 channel complexes.* Nature, 2021. **599**(7883): p. 158-164.
10. Angelova, P.R. and W.S. Müller, *Arachidonic acid potently inhibits both postsynaptic-type Kv4.2 and presynaptic-type Kv1.4 IA potassium channels.* Eur J Neurosci, 2009. **29**(10): p. 1943-50.
11. Pan, X., et al., *Structure of the human voltage-gated sodium channel Na.* Science, 2018. **362**(6412).
12. Yan, Z., et al., *Structure of the Nav1.4-beta1 Complex from Electric Eel.* Cell, 2017. **170**(3): p. 470-482 e11.
13. Sali, A. and T.L. Blundell, *Comparative protein modelling by satisfaction of spatial restraints.* J Mol Biol, 1993. **234**(3): p. 779-815.
14. de Jong, D.H., et al., *Improved Parameters for the Martini Coarse-Grained Protein Force Field.* J Chem Theory Comput, 2013. **9**(1): p. 687-97.

15. Duncan, A.L., R.A. Corey, and M.S.P. Sansom, *Defining how multiple lipid species interact with inward rectifier potassium (Kir2) channels.* Proc Natl Acad Sci U S A, 2020. **117**(14): p. 7803-7813.

16. Qi, Y., et al., *CHARMM-GUI Martini Maker for Coarse-Grained Simulations with the Martini Force Field.* J Chem Theory Comput, 2015. **11**(9): p. 4486-94.

17. Jo, S., et al., *CHARMM-GUI: a web-based graphical user interface for CHARMM.* J Comput Chem, 2008. **29**(11): p. 1859-65.

18. Abraham, M., et al., *High performance molecular simulations through multi-level parallelism from laptops to supercomputers*. 2015: SoftwareX.

19. Song, W., et al., *PyLipID: A Python Package for Analysis of Protein-Lipid Interactions from Molecular Dynamics Simulations.* J Chem Theory Comput, 2022. **18**(2): p. 1188-1201.

20. McGibbon, R.T., et al., *MDTraj: A Modern Open Library for the Analysis of Molecular Dynamics Trajectories.* Biophys J, 2015. **109**(8): p. 1528-32.

21. Corey, R.A., et al., *Insights into Membrane Protein-Lipid Interactions from Free Energy Calculations.* J Chem Theory Comput, 2019. **15**(10): p. 5727-5736.

22. Oakes, V., S. Furini, and C. Domene, *Effect of anionic lipids on ion permeation through the KcsA K.* Biochim Biophys Acta Biomembr, 2020. **1862**(11): p. 183406.

23. Poveda, J.A., et al., *Modulation of the potassium channel KcsA by anionic phospholipids: Role of arginines at the non-annular lipid binding sites.* Biochim Biophys Acta Biomembr, 2019. **1861**(10): p. 183029.

24. Chodera, J.D., *A Simple Method for Automated Equilibration Detection in Molecular Simulations.* J Chem Theory Comput, 2016. **12**(4): p. 1799-805.

25. Bennett, C., *Efficient estimation of free energy differences from Monte Carlo data.* 1976: Journal of Computational Physics. p. 245-268.
