# Supplementary material for: Cryo-EM structure of the human Kv3.1 channel reveals gating control by the cytoplasmic T1 domain
Source: Nat Commun. 2022 Jul 15;13:4087. doi: 10.1038/s41467-022-29594-w (PMC9287412; doi:10.1038/s41467-022-29594-w)
Supplement: Supplementary file 3 — Description of additional Supplementary File [file 41467_2022_29594_MOESM3_ESM.pdf]

Descriptions of Additional Supplementary data files

Supplementary Movie M1: Cryo-EM map illustrating intersubunit interactions between T1 and ATM

Overall EM map of the human Kv3.1a tetramer. The displayed map has the same color scheme as Fig. 1 A, with each subunit of the channel displayed in a different color to illustrate the domain swapping between PD and VSD and the intersubunit interactions between T1 and ATM of adjacent subunits.

Part of the micelle around the TM region is shown as semi-transparent white densities. The movie was generated in chimera

Supplementary Movie M2: Trajectory from an MD simulation at activating potential showing R4 upward movement in the S4 voltage-sensing domain of the VSD.

A single protomer of Kv3.1a is displayed as light grey cartoon with gating charges R1-R6 in S4 shown in green space-fill representation and F233 of the charge-transfer center (CTC) shown in red space-fill presentation. Over the course of the trajectory, an upward translocation of the S4 helix is observed with a significant Z-displacement of the voltage-sensing arginine R320 (R4).
